# Inulin‐Butyrate Nanogel for Modulation of Gut Microbiome, Intestinal Barrier, and Regulatory T‐Cells in Colitis

**DOI:** 10.1002/smll.202513252

**Published:** 2026-03-19

**Authors:** Nayoon Park, Bom Lee, Hyeon‐Jeong Jeon, Ji Yeon Kim, Suyeon Park, Seong‐Eun Kim, Dong‐Kyu Lee, Yonghyun Lee

**Affiliations:** ^1^ College of Pharmacy Ewha Womans University Seoul South Korea; ^2^ Gradutate Program in Innovative Biomaterials Convergence Ewha Womans University Seoul South Korea; ^3^ Department of Internal Medicine Ewha Womans University College of Medicine Seoul South Korea; ^4^ College of Pharmacy Chung‐Ang University Seoul South Korea

**Keywords:** gut microbiome, inflammatory bowel diseases, inulin‐butyrate nanogel, nanomedicine, regulatory T‐cells

## Abstract

Inflammatory bowel diseases (IBD) arise from a vicious cycle of intestinal barrier dysfunction, gut microbiome dysbiosis, and dysregulated immune responses. Current therapies predominantly suppress immunity but fail to address root causes or break this cycle. While inulin, a prebiotic, restores microbial diversity and enables colon‐targeted drug delivery, they lack specificity for inflamed tissue. On the other hand, even though butyrate, a microbial metabolite, is a potent enhancer of intestinal barrier integrity and anti‐inflammatory Treg cell differentiation, their clinical applications are limited by rapid systemic absorption, impractical dosing, and unpleasant odor. To address these limitations, we have developed an inulin‐butyrate conjugate‐based nanogel (IBN) which is capable of targeted modulation of gut microbiome, intestinal barrier, and immune systems in colitis. In dextran sodium sulfate (DSS)‐induced colitis mice, IBN specifically accumulates in the inflamed colon and released high amounts of butyrate via gut microbial enzymes (inulinase/esterase). The inulin shell improved the gut microbiome, while the released butyrate enhances intestinal barrier functions and promotes Treg differentiation, yielding robust therapeutic activity. Taken together, IBN addresses the multifactorial nature of IBD, offering a biocompatible, transformative strategy to disrupt the disease cycle and restore gut homeostasis.

## Graphical abstract

IBD is driven by a vicious cycle of dysbiosis, barrier dysfunction, and immune dysregulation. We developed an inulin–butyrate conjugate nanogel (IBN) that accumulates in inflamed colon and releases butyrate via microbial inulinase/esterase. The inulin shell remodels the microbiome, while localized butyrate strengthens barrier integrity and promotes Treg differentiation, resulting in robust therapeutic efficacy in DSS colitis.

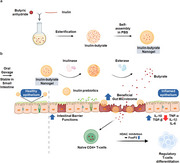

## 1 Introduction

Although the exact underlying causes of inflammatory bowel diseases (IBD) remain unclear, individuals susceptible to IBD commonly exhibit a disrupted intestinal barrier, a dysbiotic microbiome, and consequent dysregulated mucosal immune responses against the dysbiotic microbiome [1, 2, 3]. Given the cyclical interdependence of these factors, beneficial modulation of these factors simultaneously is crucial for effective IBD management. However, most current targeted therapies and conventional medications primarily focus on suppressing the dysregulated immune responses [4, 5, 6, 7, 8], thereby failing to address the root causes and break the vicious cycle driving disease progression [1, 9, 10, 11, 12].

Promoting the enhancement of the proliferation of beneficial bacteria is important to improve the dysbiosis of the gut microbiome [13]. Approaches such as fecal microbiota transplantation (FMT) and bacterial consortia demonstrate efficacy but face limitations for chronic use, including donor variability, compositional inconsistency, long‐term safety concerns, and challenges in maintaining bacterial viability [14, 15, 16]. Prebiotics, such as inulin, offer a promising alternative due to their safety, stability, and ease of administration. Inulin, a plant‐derived, water‐soluble dietary fiber, resists digestion in the stomach and the small intestine and reaches the colon intact, where it serves as a prebiotic nourishing beneficial bacteria [17, 18, 19, 20, 21]. Inulin can also be fermented by the colonic microbiota to produce short‐chain fatty acids, including butyrate [22, 23]. Its natural gelling property enables versatile formulation applications, allowing for targeted drug delivery to the colon while simultaneously restoring microbial diversity [24, 25]. However, prebiotics, including inulin, lack inflamed site specificity, modulating both healthy and inflamed regions indiscriminately [26, 27, 28]. Thus, strategies to localize their activity to inflamed sites are urgently needed.

Microbial‐derived metabolites, including short‐chain fatty acids (SCFAs) and bile acids, play a crucial role in the modulation of intestinal barrier functions and immune responses [22, 29, 30]. Butyrate, produced by commensal Clostridia, enhances colonic regulatory T (Treg) cell differentiation via histone deacetylase (HDAC) inhibition at the Foxp3 locus, suppresses inflammation in colitis [31, 32, 33]. In addition, there have been various reports that butyrate can enhance the intestinal barrier by facilitating tight junction assembly and protect the intestinal epithelium against oxidative stress‐mediated damage [34]. However, orally administered butyrate is mostly absorbed into the systemic circulation at the small intestine and only minimal amounts of butyrate reach to the colon [35, 36, 37, 38]. Indeed, as butyrate has a simple structure, it requires higher concentrations for the beneficial actions. Additionally, its unpleasant odor and volatility hinder clinical applications [39, 40, 41].

To address these limitations, we developed an inulin–butyrate conjugate nanogel (IBN), the first reported nanomaterial synthesized by covalently linking inulin and butyrate to target inflamed intestinal tissue and simultaneously modulate the gut microbiome, immune responses, and intestinal barrier in colitis. IBN selectively localizes to inflamed colonic regions while resisting premature degradation and systemic absorption, thereby enabling targeted microbiome modulation and releasing high concentrations of butyrate through the gut microbial enzymes inulinase and esterase. The butyrate released specifically at the inflamed site enhances intestinal barrier integrity and promotes Treg cell differentiation, leading to marked amelioration of colitis. Owing to its simple, biocompatible structure and robust therapeutic efficacy, IBN holds substantial transformative potential for IBD therapy.

## 2 Result

### 2.1 Preparation of Inulin‐Butyrate Conjugate‐Based Nanogel

**FIGURE 1:**
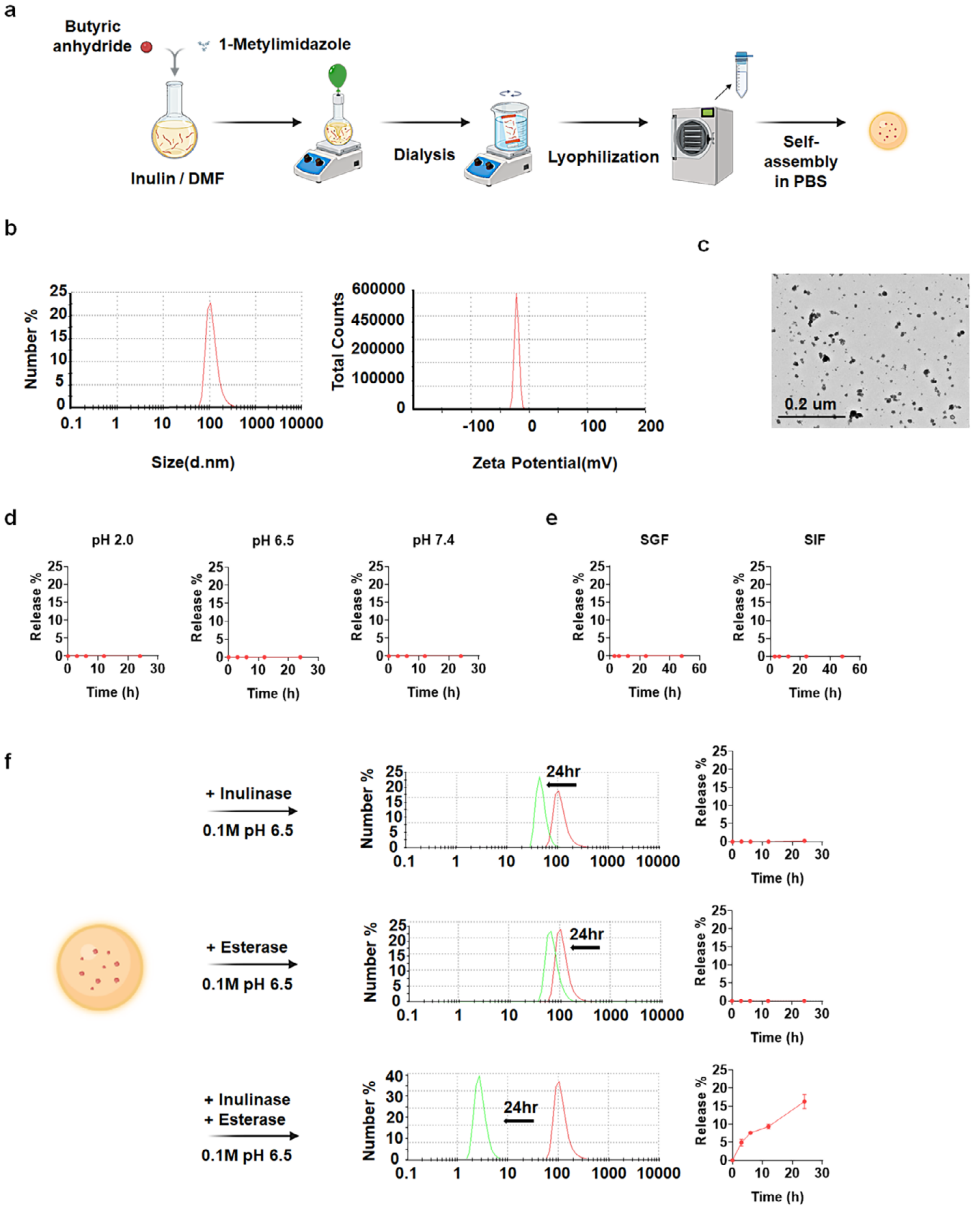
Preparation of inulin‐butyrate conjugate‐based nanogel. (a) Schematic illustration of IBN synthesis via esterification of inulin with butyric acid anhydride, followed by dialysis and lyophilization. (b) Hydrodynamic size distribution and zeta potential of IBN measured by dynamic light scattering (DLS). (c) Morphology of IBN visualized by transmission electron microscopy (TEM). (d) Drug release profiles of IBN at different pH conditions: pH 2.0, pH 6.5, and pH 7.4 over 24 h. (e) Drug release profiles of IBN in simulated gastric fluid (SGF) and simulated intestinal fluid (SIF) over 48 h. (f) Enzyme‐responsive degradation of IBN analyzed by DLS after treatment with inulinase (25 U/mg), esterase (15 U/mg), or both enzymes. DLS profiles were recorded at 0 and 24 h. Butyrate release from IBN was quantified under the same conditions at 0, 3, 6, 12, and 24 h. Scale bar, 0.2 µm C. Data are presented as mean ± s.e.m. from a representative experiment of three independent experiments. ^*^ *p* < 0.05, ^**^ *p* < 0.01, ^***^ *p* < 0.001, analyzed by one‐way ANOVA with LSD post hoc test.

We synthesized a series of inulin‐butyrate conjugate polymers with varying degrees of substitution (DS) via a simple esterification reaction from inulin and butyric anhydride (Figure 1a). Successful synthesis and DS quantification were confirmed by ^1^H NMR analysis (Figure S1). Conjugates with high DS values exhibited reduced aqueous solubility, while those with low DS failed to form stable nanostructures and had low butyrate loading percentages. Consequently, an intermediate DS value of 0.51, which corresponded to a drug loading efficacy (DLE) of 21.7 wt.%, was selected for further studies (Table S1). We confirmed that inulin‐butyrate conjugate forms a nanostructure (inulin‐butyrate nanogel, IBN) via transmission electron microscopy and dynamic light scattering (DLS) analysis. These results revealed that IBN has a size of 57.3 ± 6.4 nm in TEM and has an average hydrodynamic diameter of approximately 123.8 ± 3.6 nm with a narrow polydispersity index (PDI), indicating uniform nanoparticle distribution (Figure 1b,c). Its zeta potential value was −21.5 ± 0.2 mV.

Furthermore, the IBN displayed a hydrated gel‐like morphology in aqueous solution, confirming its self‐assembled nanogel state (Figure S2). Unlike free butyrate, IBN emitted no unpleasant odor, a key practical advantage for clinical translation. IBN also remained colloidally stable under both acidic and neutral pH conditions and in simulated gastric and small‐intestinal fluids, indicating minimal premature disassembly and negligible butyrate release before reaching the colon (Figure 1d,e, Figure S3). To evaluate enzymatic responsiveness, IBN was incubated with inulinase, esterase, or both enzymes, and nanoparticle stability was assessed via DLS (Figure 1f). Esterase alone caused negligible size changes, suggesting steric hindrance from the nanogel's hydrophobic core shielded the internally positioned ester bonds. Inulinase alone partially degraded the nanogel, reducing its size, while co‐treatment with both enzymes led to dramatic nanoparticle disintegration. Complementary HPLC analysis of butyrate release (Figure 1f) revealed minimal liberation with esterase alone (due to restricted ester bond accessibility) and none with inulinase alone. Notably, co‐treatment with both enzymes accelerated butyrate release, probably owing to a sequential mechanism: inulinase disrupts the nanogel structure, enabling esterase to hydrolyze exposed ester bonds. The combination of gastrointestinal stability and this dual‐enzyme synergy enables inflamed colon‐specific butyrate delivery, as inulinase—produced exclusively by the gut microbiota in the colon—ensures enzymatic activation occurs only at the inflamed colon, preventing premature release in the small intestine. Finally, butyrate quantification in both feces and colonic tissue revealed that whereas DSS alone markedly reduced these levels, IBN treatment in DSS‐colitis mice significantly increased butyrate concentrations in both compartments, further supporting the inflamed‐colon‐specific release of butyrate from IBN. (Figure S4)

### 2.2 IBN Accumulates in Inflamed Colon

**FIGURE 2:**
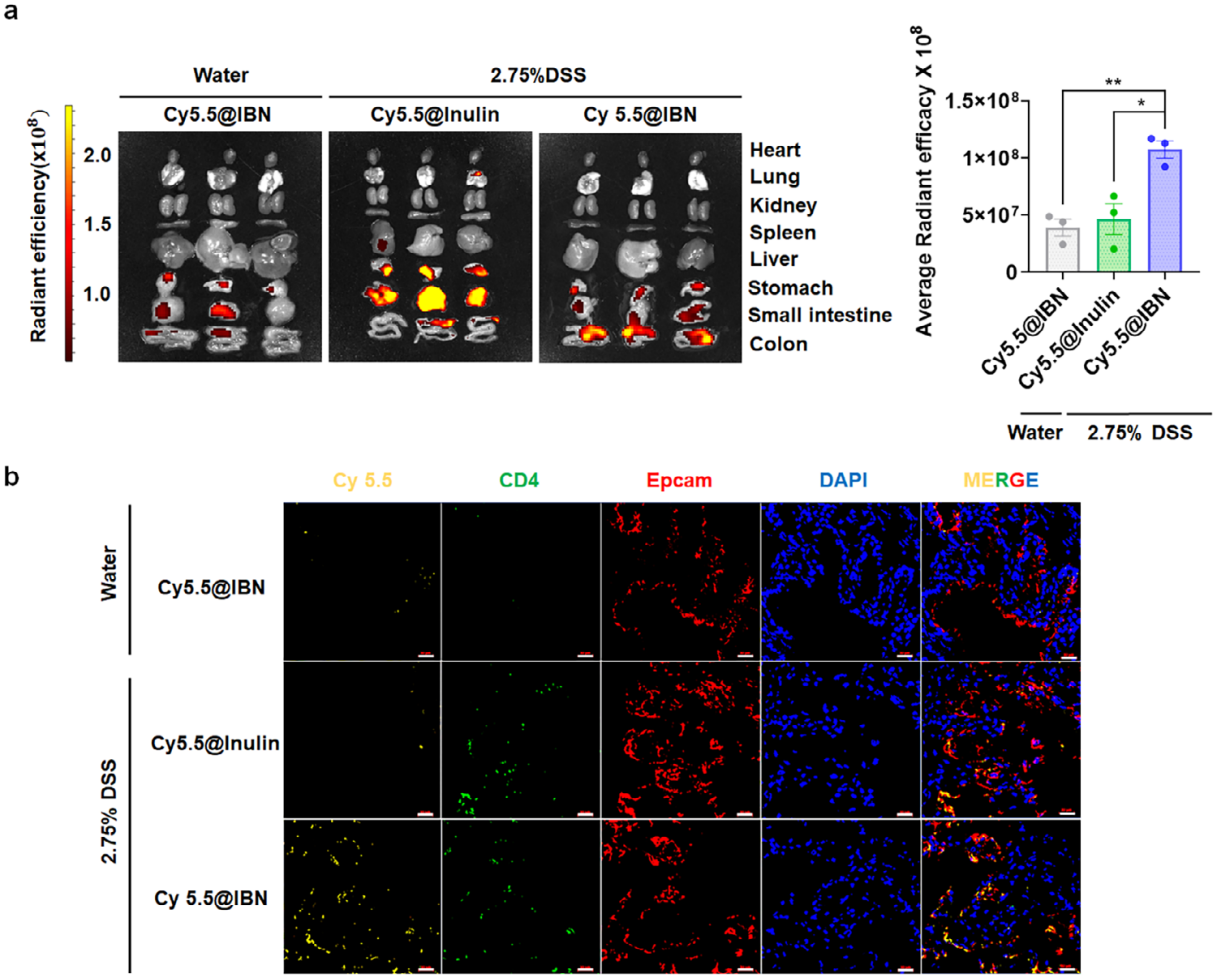
IBN accumulates in the inflamed colon. (a) After 10 h of treating animals with Cy5.5@IBN, Cy5.5@Inulin (20 mg/dose, equivalent Cy5.5 dose), their organs were imaged by an in vivo imaging system (IVIS) and quantified for Cy5.5 fluorescence signal. (b) Healthy or DSS‐colitis mice were orally administered on day 7 with Cy5.5@IBN, Cy5.5@Inulin (20 mg/dose, equivalent Cy5.5 dose), and colon tissues were excised after 10 h, stained with anti‐CD4 and anti‐EpCAM antibodies and visualized by confocal microscopy. Scale bars, 20 µm (b). Data are presented as mean ± s.e.m. from a representative experiment (*n* = 3 biologically independent animals). ^*^ *p* < 0.05, ^**^ *p* < 0.01, ^***^ *p* < 0.001, analyzed by one‐way ANOVA with LSD post hoc test.

Given the reported ability of orally administered nanoparticles to preferentially accumulate at inflamed sites [42] through the leaked intestinal barrier, we assumed that IBN might also have the ability to target inflamed regions in the colon. To verify this, IBN loaded with Cy5.5 or inulin loaded with Cy5.5 was orally administered in mice given 2.75% DSS or normal water. Notably, IBN loaded with Cy5.5 specifically localizes to the inflamed colon of mice given 2.75% DSS, but Cy5.5 signals were negligibly detected in the healthy colon of mice given normal water (Figure 2a). In contrast, inulin loaded with Cy5.5 did not accumulate in the inflamed colon (Figure 2a). These findings suggest that IBN has the ability to selectively target inflamed sites in the colon.

We next assessed whether an IBN‐associated molecule can preferentially localize to inflamed colonic cell populations. In the colon, Cy5.5@IBN produced stronger fluorescence signals in the intestinal epithelium (EpCAM^+^) and CD4^+^ T cells than Cy5.5@inulin (Figure 2b). These results indicate that IBN enables preferential accumulation/association of its loaded cargo with inflamed colonic epithelium and CD4^+^ T cells.

### 2.3 IBN Exhibits Strong Therapeutic Activity in a Murine DSS‐induced Colitis Model

**FIGURE 3:**
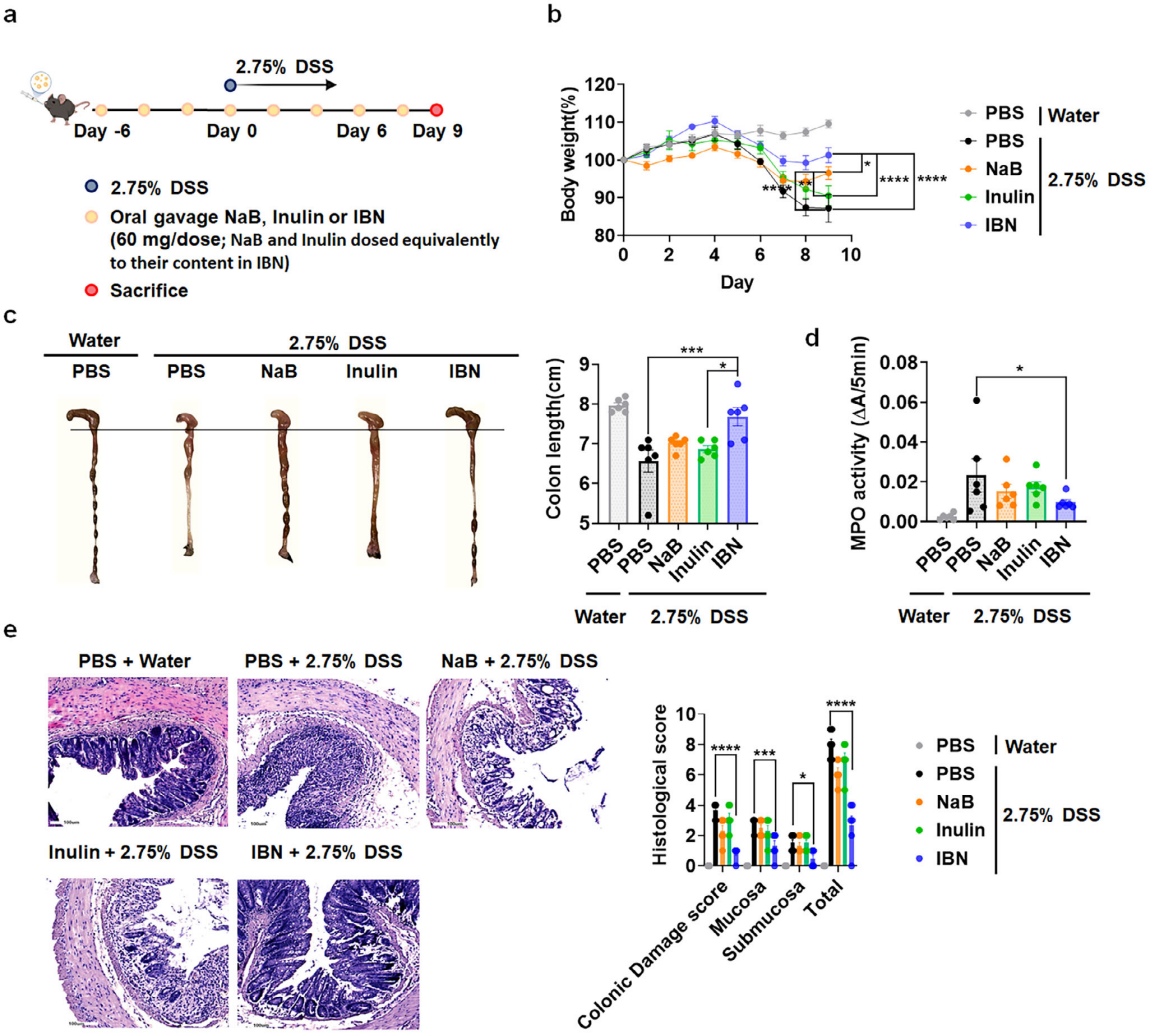
IBN exhibit strong therapeutic activity in a murine DSS‐induced colitis model. (a) C57BL/6 mice were provided with water or 2.75% DSS‐containing water for 6 days. On days ‐6, ‐4, ‐2, 0, 2, 4, 6 and 8 mice were orally administered with PBS, NaB (equivalent to their content in IBN), Inulin (equivalent to their content in IBN) or IBN (60 mg/dose). (b) Daily bodyweight changes in each group for 9 days. (c–e), On day 9, animals were euthanized, and (c) colon length, (d) colonic MPO activity, and (e) colonic damage scores were measured. Scale bars, 100 µm (e). Data are presented as mean ± s.e.m. from a representative experiment (*n* = 6 biologically independent animals) from independent experiments. ^*^ *p* < 0.05, ^**^ *p* < 0.01, ^***^ *p* < 0.001, ^****^ *p* < 0.0001, analyzed by (c, d, e) one‐way or (b) two‐way ANOVA followed by LSD post hoc test.

We next evaluated the therapeutic efficacy of orally administered IBN in a murine DSS‐induced colitis model (Figure 3a, Figure S6a). Preliminary comparisons revealed that inulin, which modulates the gut microbiome, outperformed resistant starch (another microbiome‐modulating agent) in mitigating DSS‐induced body weight loss in DSS‐induced colitis mice (Figure S5). Orally administered IBN significantly ameliorated bodyweight loss, suppressed disease activity, and attenuated inflammation‐associated reduction of colon length in DSS‐colitis mice compared to inulin, butyrate and inulin/butyrate mixture groups (Figure 3b‐–d, Figure S6b,c). Furthermore, IBN administration markedly decreased myeloperoxidase (MPO) activity, preserved colonic epithelial integrity, and reduced immune cell infiltration in inflamed colon tissue of DSS‐colitis mice, compared to inulin and butyrate treatment alone (as reflected by the reduced histological scores based on epithelial damage/crypt distortion and inflammatory cell infiltration) (Figure 3e,f, Figure S6d,e). Comparable efficacy was observed when IBN treatment was started on day 0 (six doses), compared with the primary regimen initiated on day −6 (eight doses) (Figure S7). These results demonstrate that IBN has a dramatic therapeutic activity when compared to inulin, which is limited to gut microbiome modulation, and butyrate, which undergoes rapid systemic absorption with minimal retention in the colon. Notably, IBN did not trigger any overt signs of systemic toxicity, autoimmunity, or pathologies in the major organs (Figure S8).

### 2.4 IBN's Gut Microbiome Modulation Activity Is Crucial in IBN Therapy

Next, we examined the mechanism underlying the therapeutic activity of IBN. We assumed that inflamed site‐targeting IBN may have gut microbiome modulation activity owing to the inulin shell, and inflamed site‐targeted release of butyrate may contribute to immune homeostasis via Treg induction and enhancement of the intestinal barrier.

**FIGURE 4:**
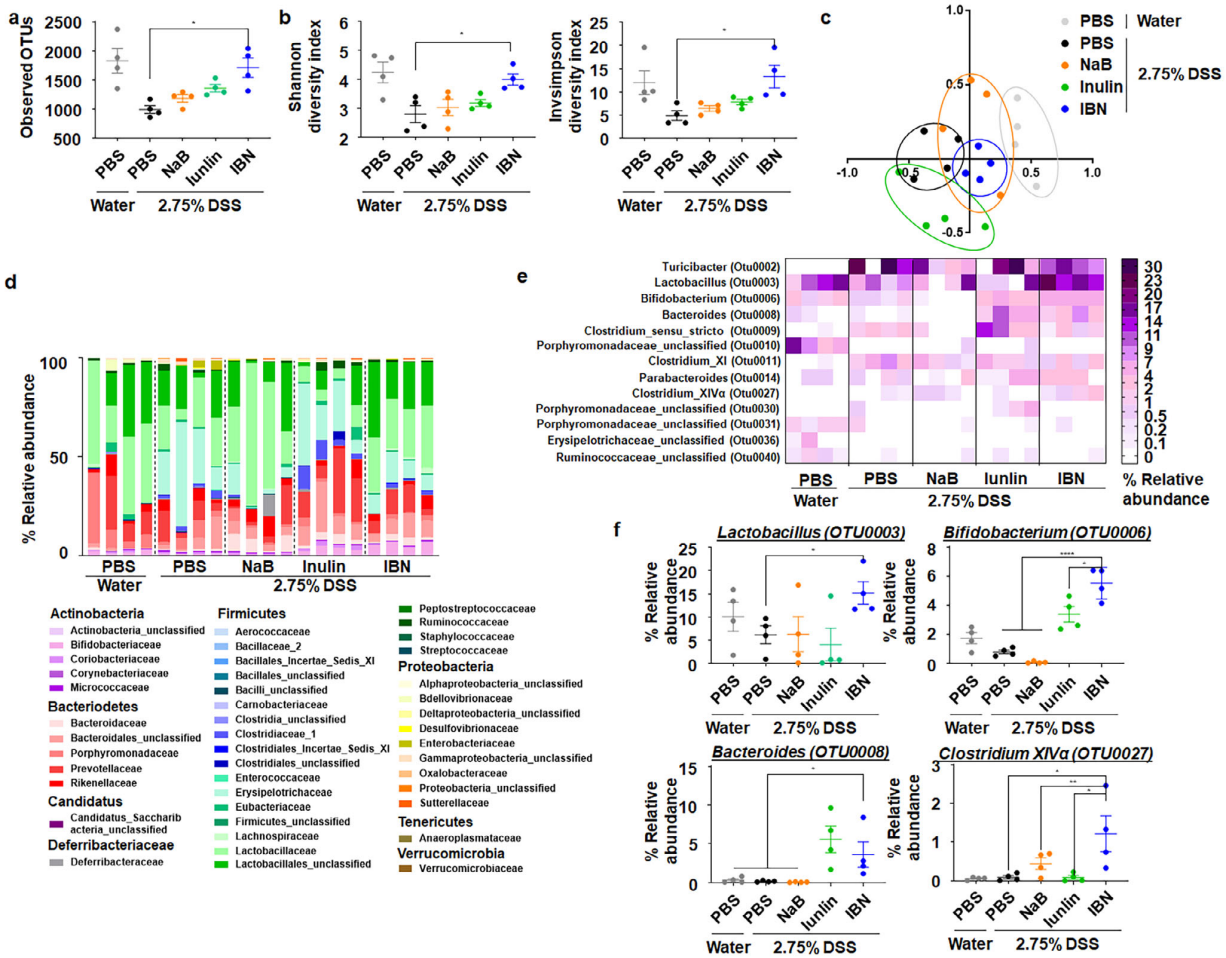
IBN modulates gut microbiome. (a–f) C57BL/6 mice were given 2.75% DSS in drinking water for 6 d and treated as shown in Figure 3. Fecal samples collected on day 9 were analyzed for gut microbiome composition by 16S rRNA gene sequencing in the V4 region. (a–b) Microbiome richness (Observed OTUs, a) and α‐diversity (Shannon and Inverse Simpson indices, b). (c) Non‐metric multidimensional scaling (NMDS) plot illustrating β‐diversity, each point denotes one mouse based on a subsample of 14,085 OTUs. d, Relative abundance of gut microbiota at the phylum and family levels (% of total sequences). (e) Heatmap presenting the relative abundance of predominant families (rows) for each mouse (columns). (f) Relative abundance of selected taxa. Data are presented as mean ± s.e.m. from a representative experiment (*n* = 4 biologically independent animals) from independent experiments. ^*^ *p* < 0.05, ^**^ *p* < 0.01, ^***^ *p* < 0.001, ^***^ *p* < 0.0001, analyzed by one‐way ANOVA with LSD multiple‐comparison post hoc test.

16s rRNA gene sequencing of the V4 region in the fecal samples showed that DSS supplementation markedly reduced bacterial richness and diversity, whereas IBN treatment significantly restored both parameters (Figure 4a,b). Non‐metric multidimensional (NMDS) plots further revealed that the gut microbiome profiles of DSS‐colitis mice treated with IBN clustered apart from those of DSS‐colitis mice given PBS, NaB, or Inulin, and were close to profiles of healthy mice (Figure 4c). At the phylum/family/genus levels, IBN markedly increased *Lactobacillus* [43, 44, 45]*, Bifidobacterium* [46, 47]*, Bacteroides* [48, 49], and *Clostridium XIVα * [50], all of which are associated with anti‐inflammation activity (Figure 4d–f). Notably, Inulin alone, a prebiotic, also enriched *Bifidobacterium* and *Bacteroides*, while NaB alone had a minimal activity on these taxa.

**FIGURE 5:**
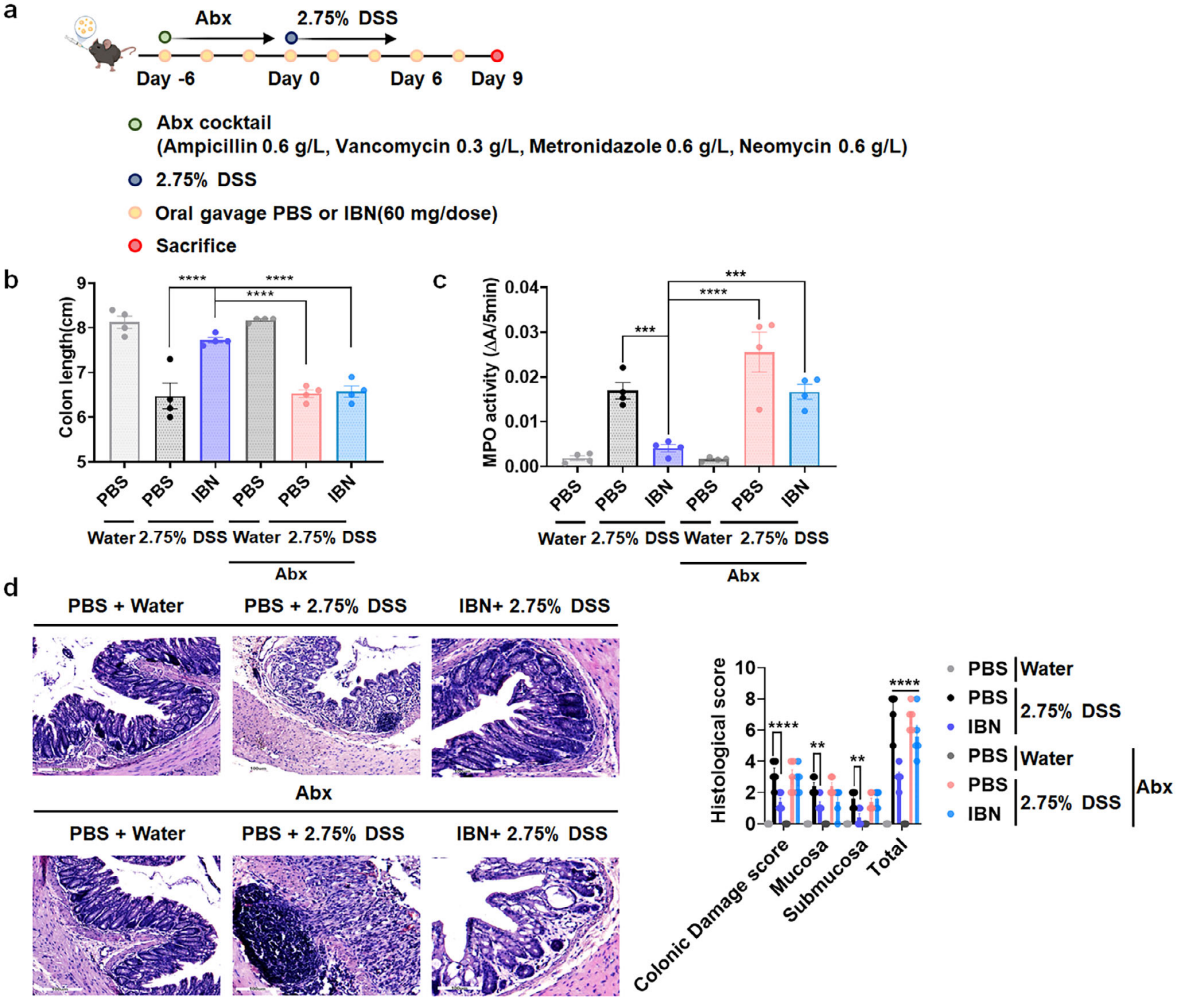
IBN's gut microbiome modulation activity is crucial in IBN therapy. (a) C57BL/6 mice were pretreated for 6 days with an antibiotic cocktail (ampicillin, vancomycin, metronidazole, and neomycin) in the drinking water, and then given either water or 2.75% DSS‐containing water for 6 days. On days −6, −4, −2, 0, 2, 4, 6, and 8, mice were orally administered with PBS or IBN (60 mg/dose). (b–d) On day 9, animals were euthanized and (b) colon length, (c) colonic MPO activity, and (d) histological damage scores were evaluated. Scale bars, 100 µm (d). Data are presented as mean ± s.e.m. from a representative experiment (*n* = 4 biologically independent animals) from independent experiments. ^*^ *p* < 0.05, ^**^ *p* < 0.01, ^***^ *p* < 0.001, ^***^ *p* < 0.0001, analyzed by one‐way ANOVA with LSD multiple‐comparison post hoc test.

To determine whether gut microbiome modulation is causally involved in the therapeutic activity of IBN, pseudo germ‐free mice was prepared by receiving an antibiotic cocktail in drinking water for six days prior to DSS administration and then, IBN or PBS was orally administered with normal mice or pseudo germ‐free mice given 2.75% DSS or normal water. Surprisingly, therapeutic activity of IBN significantly abrogated in pseudo germ‐free mice, indicative of crucial role of gut microbiome in IBN therapy. (Figure 5).

### 2.5 IBN Promotes Regulatory T‐cells and Enhances Intestinal Barrier Functions

**FIGURE 6:**
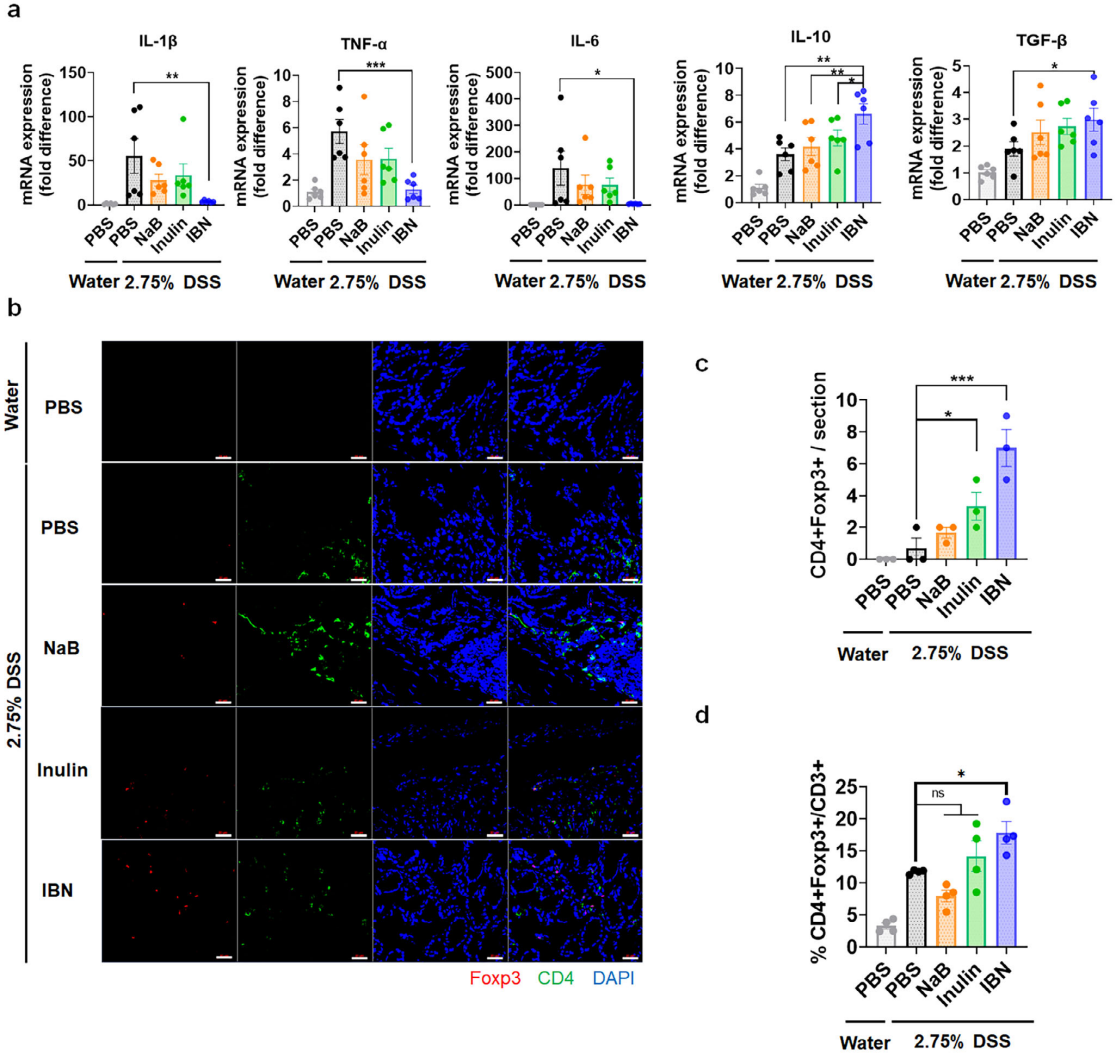
IBN promotes regulatory T‐cells. (a‐d), Healthy or DSS‐colitis mice were orally administered on day −6. −4, −2, 0, 2, 4, 6 and 8 mice were orally administered with PBS, NaB (equivalent to their content in IBN), Inulin (equivalent to their content in IBN) or IBN (60 mg/dose). Colon tissues were excised and analyzed for the mRNA levels of pro‐inflammatory and anti‐inflammatory cytokines (a) and colonic CD4+Foxp3+ Treg cells populations (b) with quantifications of CD4+Foxp3+ cells in immunofluorescence images (c) and flow cytometry (d). Scale bars, 20 µm. Data are presented as mean ± s.e.m. from a representative experiment (*n* = 6 for (a), *n* = 3 for (b) and (c), *n* = 4 for (d) biologically independent animals per group). ^*^ *p* < 0.05, ^**^ *p* < 0.01, ^***^ *p* < 0.001, ^****^ *p* < 0.0001, analyzed by two‐way ANOVA (b) or one‐way ANOVA (c–e) followed by LSD post hoc test.

**FIGURE 7:**
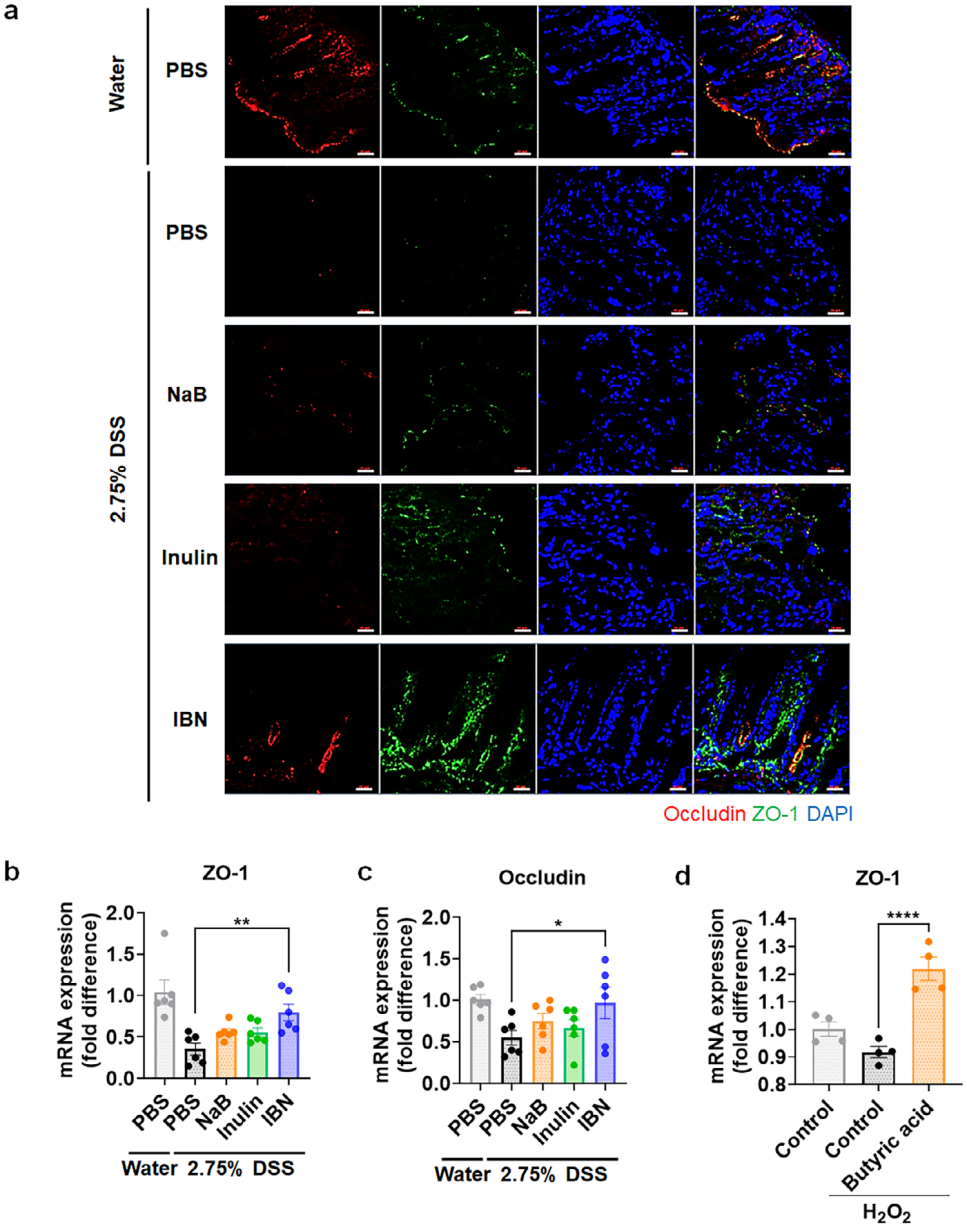
IBN enhances intestinal barrier functions. (a‐c) Healthy or DSS‐colitis mice were orally administered on day ‐6. ‐4, ‐2, 0, 2, 4, 6 and 8 mice were orally administered with PBS, NaB (equivalent to their content in IBN), Inulin (equivalently to their content in IBN) or IBN (60 mg/dose). Colon tissues were excised and analyzed for the expression patterns (a) and mRNA levels (b, c) of ZO‐1 and occludin‐1. (d) mRNA expression levels of ZO‐1 in HCT‐116 cells treated with control medium or butyrate (0.5 mM) in the presence or absence of hydrogen peroxide (200 µm). Scale bars, 20 µm (a). Representative images from three slides are shown, obtained from *n* = 6 biologically independent animals across independent experiments. Data are presented as mean ± s.e.m. ^*^ *P* < 0.05, ^**^ *P* < 0.01, ^***^ *P* < 0.001 and ^****^ *P* < 0.0001, analyzed by one‐way ANOVA followed by LSD post hoc test.

Since released butyrate has the ability to promote regulatory T‐cells [31, 32, 33] and enhance intestinal barrier function [51, 52], we then evaluated the immunomodulatory activity of IBN. IBN treatment significantly reduced local levels of pro‐inflammatory cytokines such as IL‐1beta, TNF‐alpha, and IL‐6 while increasing IL‐10 and TGF‐beta in DSS‐colitis mice. However, inulin and butyrate treatment have moderate activity (Figure 6a). Notably, IBN treatment significantly increased populations of CD4+Foxp3+ Treg cells compared to inulin and butyrate treatment alone (Figure 6b–d, Figure S9), probably owing to the HDACi activity of released butyrate [31, 32, 33]. In DSS‐induced colitis mice, oral administration of IBN restored normal expression and mRNA levels of the tight junction proteins ZO‐1 and occludin‐1—critical for maintaining intestinal barrier integrity (Figure 7a,b), probably due to butyrate‐mediated enhancement of intestinal barrier functions (Figure 7c). In contrast, neither inulin nor butyrate alone significantly improved tight junction regulation. These findings demonstrate that IBN modulate immune homeostasis and strengthens the intestinal barrier functions owing to the inflamed site‐specific release of butyrate.

## 3 Discussion

**FIGURE 8:**
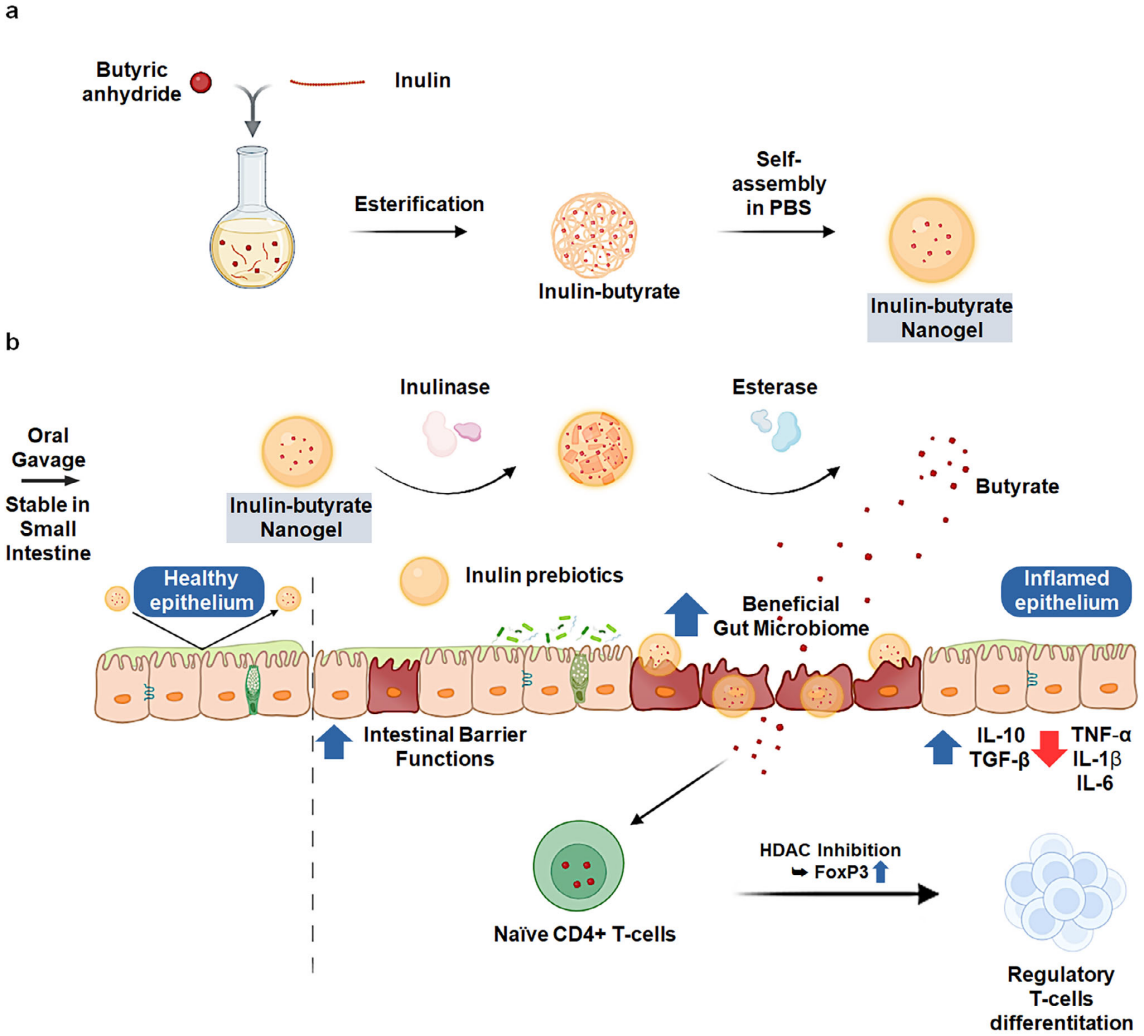
Orally administered inulin‐butyrate nanogel (IBN) accumulates in the inflamed regions and modulates gut microbiome, intestinal barrier and regulatory T‐cells in colitis. (a) A schematic of inulin‐butyrate conjugation from butyric anhydride and inulin via simple esterification and formation of nanogels from the inulin butyrate conjugate. (b) Inulin‐butyrate conjugate‐based nanogel specifically accumulates in the inflamed colon and releases butyrate via gut microbial enzymes, inulinase and esterase while minimizing absorption and degradation in the small intestine. Thereby, inulin could beneficially modulate the gut microbiome and released butyrate could enhance intestinal barrier functions and promote regulatory T‐cells. This leads to amelioration of inflammatory bowel diseases.

Inflammatory bowel diseases (IBD) result from a vicious cycle involving intestinal barrier dysfunction, gut microbiome dysbiosis, and dysregulated immune responses [1, 2]. Current therapies primarily suppress the immune system but fail to break this cycle [9, 10, 11]. To address this gap, we developed IBN as a targeted nanomedicine designed to modulate the gut microbiota, restore barrier function, and regulate immune responses (Figure 8). IBN, with a size of approximately 123.8 ± 3.nm and a narrow PDI, exhibited gel‐like properties and lacked any unpleasant odor. In a murine DSS‐induced colitis model, orally administered IBN selectively accumulated in the inflamed colon—especially within CD4+ T cells and the inflamed epithelium—while showing minimal presence in healthy tissue (Figure 2). Notably, IBN rapidly released free butyrate upon co‐treatment with esterase and inulinase, whereas esterase or inulinase alone had minimal effects, respectively (Figure 1). Since inulinase is absent in the small intestine and present only in the large intestine, and IBN specifically accumulates at inflamed sites within the colon, IBN enables inflamed colon‐specific degradation by gut microbial enzymes, ensuring localized butyrate release after reaching the inflamed colon, with minimal premature release in the small intestine. At the inflamed site, the inulin shell may act as a prebiotic to modulate the gut microbiome (Figure 4), while the released butyrate improves intestinal barrier functions (Figure 7) and promotes Foxp3+ regulatory T cell differentiation (Figure 6). Finally, IBN exhibited potent therapeutic efficacy in the colitis model (Figure 3, Figures S6 and S7) without any systemic toxicity (Figure S8). Importantly, the therapeutic activity of IBN was significantly abrogated by antibiotic pretreatment, highlighting the crucial role of the gut microbiome in IBN therapy (Figure 5).

Inulin can modulate the gut microbiome, however, it lacks inflamed‐site specificity and therefore acts as a broad, non‐targeted prebiotic throughout the colon. The moderate immunomodulatory and barrier‐protective effects observed with inulin alone may, at least in part, arise from microbiota‐mediated fermentation in the colon that generates endogenous SCFAs, including butyrate. In contrast, free butyrate is directly bioactive but is rapidly absorbed in the upper GI tract, limiting its delivery and retention in the inflamed colon. By contrast, IBN localizes the inulin shell to inflamed lesions, leading to a distinct microbiome modulation together with localized butyrate availability, thereby supporting enhanced intestinal barrier function and promoting regulatory T‐cell differentiation. Collectively, this enables more effective coordinated regulation of the gut microbiome, mucosal immune homeostasis, and intestinal barrier function than inulin or free butyrate alone.

In conclusion, we developed an inulin–butyrate‐based nanogel by simply conjugating the prebiotic inulin with the microbial metabolite butyrate, enabling targeted modulation of the gut microbiome, intestinal barrier, and immune system—an effect that is difficult to achieve with either component alone. As inulin is a natural compound and butyrate is an endogenous microbiome‐mediated metabolite, IBN exhibits high biocompatibility. Furthermore, IBN has a simple composition, based solely on the inulin–butyrate conjugate, along with effective masking of butyrate's unpleasant odor. Thus, these interesting features of IBN support its strong potential for clinical translation. Given the central role of dysbiotic gut microbiome and disrupted intestinal barrier in various systemic diseases, our findings highlight the potential of IBN as a versatile platform for treating not only colitis but other microbiome‐related disorders.

## 4 Experimental Section

### 4.1 Synthesis of Inulin‐Butyrate Conjugate

To synthesize inulin‐butyrate conjugate, 20 mg of inulin (Sigma–Aldrich) was dissolved in 0.1 mL of degassed N,N‐dimethylformamide (DMF, Fisher Scientific). After complete dissolution, 3.58 µL of 1‐methylimidazole (1‐MI, Sigma–Aldrich) and 9.4 µL of butyric anhydride (Alfa Aesar) were added to the solution. The mixture was stirred at 40°C for 24 h under a nitrogen atmosphere to facilitate esterification between inulin and butyric anhydride. Upon completion, the reaction mixture was dialyzed using a 1 kDa MWCO regenerated cellulose (RC) membrane (Spectra/Por 6, Spectrum Laboratories) against distilled water for 2 days to remove residual DMF, 1‐MI, and unreacted butyric anhydride. The resulting solution was lyophilized to obtain the inulin‐butyrate conjugate. To confirm the chemical structure of the product, the lyophilized butyrlated inulin was dissolved in 1 mL of deuterium oxide (D2O), and the solution was transferred to a nuclear magnetic resonance (NMR) tube. The ^1^H‐NMR spectra were acquired using a 400 MHz spectrometer. The degree of substitution (DS) was quantified from the ^1^H NMR spectra by comparing the integrated peak area of the butyrate protons (δ 0.8–2.5 ppm) with that of the inulin backbone (fructose‐ring) protons (δ 3.5–4.4 ppm). The drug loading capacity (DLC) was then calculated from the DS value as the theoretical butyric acid‐equivalent content, assuming complete hydrolysis of the ester linkages, using the following equation.Sigma‐AldrichSigma‐AldrichDrug loading capacity (DLC, %) = [DS × MW_Butyric acid_] / [MW_Polysaccharides unit_ + DS × (MW_Butyric acid_—MW_water_)] × 100

### 4.2 Preparation of Inulin‐Butyrate Nanogels(IBN)

After dissolving 5 mg of inulin‐butyrate conjugate in 1 mL of chloroform, the solvent was evaporated under nitrogen gas to form a thin film. Subsequently, 1 mL of filtered distilled water was added, followed by ultrasonication for 10 min. The resulting suspension was filtered through a 0.45 µm membrane to obtain IBN. The morphology of IBN was examined using transmission electron microscopy (TEM, JEM‐2100F, JEOL, Japan). The hydrodynamic diameter and zeta potential of IBN were measured using dynamic light scattering (DLS, Zetasizer Nano‐ZS90, Malvern Instruments, UK). IBN was diluted in phosphate‐buffered saline(PBS) for subsequent experiments.

### 4.3 Preparation of Cy5.5@IBN

Cy5.5‐loaded IBN was prepared using a thin‐film hydration method. Briefly, 20 mg of IBN and 0.2 mg of Cy5.5‐COOH were dissolved in 1 mL of chloroform in a 2 mL microcentrifuge tube (Eppendorf). The organic solvent was evaporated under a gentle nitrogen stream, forming a thin film along the inner wall of the tube. The film was then hydrated with 1 mL of filter‐sterilized PBS, followed by 10 min of sonication to facilitate nanoparticle formation. The resulting suspension was centrifuged at 3,000 × g for 5 min to remove unincorporated Cy5.5. The supernatant was collected and dialyzed against PBS using a 1 kDa MWCO membrane for 24 h to remove free or loosely associated dye and retain only strongly entrapped Cy5.5 for imaging. The Cy5.5 loading efficiency was determined by measuring the absorbance of the nanoparticle solution at the maximum absorption wavelength of Cy5.5 using a UV–vis spectrophotometer. For comparison, a control formulation using free inulin was prepared under identical conditions. Encapsulation efficiency (%) = (weight of drug in particle/weight of drug added initially) x 100.

Drug loading percentage (%) = [weight of drug in particle/(weight of drug in particle + weight of IBN added initially)] x 100.

### 4.4 pH‐Dependent Butyrate Release from IBN

In vitro butyrate release was evaluated under varying pH without enzyme to rule out the effect of pH. IBN was dispersed in 0.1 M phosphate buffer of pH 2.0, 6.5 and PBS of pH 7.4. The reaction was carried out in microcentrifuge tubes containing IBN (equivalent to 50 mm butyrate), and incubated at 37°C with gentle shaking (100 rpm) for up to 24 h. At each time point, the solution was collected and filtered through a 0.22 µm syringe filter. The concentration of released butyrate was quantified using high‐performance liquid chromatography (HPLC) equipped with a UV detector. Separation was performed on a C18 column using an isocratic mobile phase composed of 90% 20 mm phosphate buffer (pH 2.3) and 10% methanol containing 0.1% trifluoroacetic acid (TFA), at a flow rate of 1.0 mL/min. Detection was carried out at 210 nm. A standard curve constructed using authentic sodium butyrate was used for quantification.

### 4.5 Butyrate Release Assessment of IBN In Simulated Gastrointestinal Fluids

In vitro butyrate release was evaluated under simulated gastric fluid (SGF) and simulated intestinal fluid (SIF). SGF was prepared by dissolving 34.2 mm of NaCl and 3.2 g/L of pepsin (2500 IU/mg, Sigma‐Aldrich) in pH 2.0 HCl solution. SIF was prepared by neutralizing 6.8 g/L KH2PO4 solution to pH 6.8 with 0.2 M NaOH solution and dissolving 1.25 g/L of pancreatin (8 × USP specifications). IBN was dispersed in SGF and SIF at a concentration corresponding to 50 mm butyrate equivalents (i.e., 50 mm with respect to conjugated butyrate) and incubated at 37°C with gentle shaking (100 rpm) for up to 48 h. At each time point, the solution was filtered through 10 kDa ultracentrifugal filter by centrifuging at 5000 × g for 10 min and the filtrate was collected for butyrate quantification. The concentration of released butyrate in filtrate was quantified using HPLC equipped with a UV detector. Separation was performed on a C18 column using an isocratic mobile phase composed of 90% 20 mM phosphate buffer (pH 2.3) and 10% methanol containing 0.1% TFA, at a flow rate of 1.0 mL/min. Detection was carried out at 210 nm. A standard curve constructed using authentic sodium butyrate was used for quantification.

### 4.6 Enzyme‐Responsive Degradation and Butyrate Release of IBN

In vitro butyrate release was evaluated under simulated intestinal conditions by dispersing IBN (equivalent to 50 mm butyrate) in 0.1 m phosphate buffer (pH 6.5) and incubating it with inulinase (0.8 mg/5 mL, 25 U/mg, Sigma‐Aldrich) and esterase (16.67 mg/ 5 mL, 15 U/mg, Sigma–Aldrich), under conditions adapted from previous reports with modifications [53, 54]. The enzymatic reaction was carried out in microcentrifuge tubes containing IBN at a concentration of 50 mm, and incubated at 37°C with gentle shaking (100 rpm) for up to 24 h. At each time point, the reaction was terminated by heating at 93 °C for 7 min, followed by centrifugation at 20,000 × g for 10 min at 4°C. The resulting supernatant was collected and filtered through a 0.22 µm syringe filter. The concentration of released butyrate was quantified using HPLC equipped with a UV detector. Separation was performed on a C18 column using an isocratic mobile phase composed of 90% 20 mm phosphate buffer (pH 2.3) and 10% methanol containing 0.1% TFA, at a flow rate of 1.0 mL/min. Detection was carried out at 210 nm. A standard curve constructed using authentic sodium butyrate was used for quantification.

### 4.7 Animals

All work performed on animals was in accordance with and approved by the Institutional Animal Care and Use Committee (IACUC) at Ewha Womans University (EWHA IACUC 21‐068‐t). All animals were obtained from the Young bio (Suwon, South Korea) as mixed littermates and housed under pathogen‐free conditions in the animal facility at Ewha Womans University. The investigators were not blinded to allocation during experiments and outcome assessment, unless each section particularly included any blind assessment.

### 4.8 IVIS Imaging

To evaluate the inflammation‐targeting capability of IBN, an acute colitis model was established by administering 2.75% (w/v) dextran sodium sulfate (DSS, Thermo Fisher Scientific) in the drinking water of 6‐week‐old female C57BL/6 mice for 6 consecutive days. On day 6, the mice were randomly assigned to the following groups: (1) healthy mice orally administered Cy5.5@IBN (20 mg/dose, containing 0.1 mg/kg of Cy5.5), (2) DSS‐induced colitis mice orally administered Cy5.5‐labeled free inulin (20 mg/dose, containing 0.1 mg/kg of Cy5.5), and (3) DSS‐induced colitis mice orally administered Cy5.5@IBN (20 mg/dose, containing 0.1 mg/kg of Cy5.5). At 10 h post‐administration, mice were euthanized and the entire colon was excised. Fluorescence signals in the excised tissues were captured using the In Vivo Imaging System (IVIS) Lumina (PerkinElmer) equipped with a Cy5.5 filter set. The exposure time was set to 5 s for all acquisitions. Fluorescence intensity in the colonic tissues was quantified using Living Image software (PerkinElmer).

### 4.9 DSS‐Induced Model of Colitis

Healthy female C57BL/6 mice (6 weeks old, 18–20 g) were randomly assigned to six groups (*n* = 6 per group) as follows: (1) PBS + normal drinking water (healthy control), (2) PBS + 2.75% DSS (colitis control), (3) NaB (at a dose equivalent to its content in IBN) + 2.75% DSS, (4) inulin (at a dose equivalent to its content in IBN) + 2.75% DSS, (5) NaB + inulin (each at a dose equivalent to their respective content in IBN) + 2.75% DSS, and (6) IBN (60 mg/dose) + 2.75% DSS. Colitis was induced by administering 2.75% (w/v) DSS in the drinking water for 6 consecutive days, followed by normal drinking water for the remainder of the experimental period. Treatment groups received oral administration of their respective formulations on days −6, −4, −2, 0, 2, 4, 6, and 8 during the DSS exposure and recovery phases. In antibiotic depletion experiments, to assess the role of gut microbiota, mice were pretreated with a broad‐spectrum antibiotic cocktail containing ampicillin (0.6 g/L), vancomycin (0.3 g/L), neomycin (0.6 g/L), and metronidazole (0.6 g/L) in drinking water for 6 days prior to DSS induction. Body weight was monitored daily throughout the experiment. Fecal samples were collected on day 9 for microbiome analysis. On the final day, mice were euthanized and the entire colon was excised. The colon length was measured, and the colon was gently washed with physiological saline. Two distal segments (0.5 cm each) were collected for histological and immunofluorescence analysis, while the remaining tissue was used for myeloperoxidase (MPO) activity assays and cytokine quantification.

### 4.10 Histology

Colon tissue samples were fixed in 4% paraformaldehyde (PFA, pH 7.4, Thermo Fisher Scientific), dehydrated through a graded ethanol series, and embedded in paraffin. Sections (5 µm thick) were prepared and stained with hematoxylin and eosin (H&E) using a commercial staining kit (Abcam) according to the manufacturer's instructions. Histopathological evaluation was performed in a blinded manner according to a previously published scoring method [55], assessing epithelial integrity, crypt architecture, and inflammatory cell infiltration. Briefly, colonic damage was assigned scores as follows: 0, normal, 1, hyperproliferation, irregular crypts, and goblet cell loss, 2, mild to moderate crypt loss (10–50%), 3, severe crypt loss (50–90%), 4, complete crypt loss, surface epithelium intact, 5, small‐ to medium‐sized ulcers (<10 crypt widths), 6, large ulcers (≥10 crypt widths). Inflammatory cell infiltration was scored separately for the mucosa (0, none, 1, mild, 2, moderate, 3, severe), submucosa (0, normal, 1, mild to moderate, 2, severe), and muscle/serosa (0, normal, 1, moderate to severe). Scores for epithelial damage and inflammatory cell infiltration were summed, resulting in a total scoring range of 0–12.

### 4.11 MPO Activity Assay

For MPO activity measurement, colon tissue was homogenized at a 1:10 (w/v) ratio in 50 mm phosphate buffer (pH 6.0) using a TissueLyser (Qiagen). The resulting homogenate was further diluted 10‐fold with 50 mm phosphate buffer containing 0.5% hexadecyltrimethylammonium bromide (HTAB, Sigma‐Aldrich). After 10 s of sonication, the suspension underwent three freeze–thaw cycles, followed by centrifugation at 20,000 × g for 20 min at 4°C. Subsequently, 5 µL of each supernatant was added to 145 µL of 50 mm phosphate buffer (pH 6.0) containing 0.15 mg/mL o‐dianisidine dihydrochloride (Sigma–Aldrich) and 0.005% H_2_O_2_. Changes in absorbance at 460 nm were recorded over 5 min. MPO activity was calculated from the absorbance readings and normalized to the total protein concentration, which was determined using the Micro BCA Protein Assay Kit (Thermo Fisher Scientific).

### 4.12 Quantitative Real‐Time PCR Analysis

To evaluate gene expression related to inflammation and intestinal barrier function, colon tissue samples were collected, homogenized, and processed for RNA extraction using the RNeasy Mini Kit (QIAGEN) according to the manufacturer's instructions. Total RNA concentration and purity were assessed using a NanoDrop spectrophotometer (Thermo Fisher Scientific). For cDNA synthesis, 1 µg of total RNA was reverse‐transcribed using the amfiRivert cDNA Synthesis Platinum Master Mix (GenDEPOT) following the manufacturer's protocol. Quantitative real‐time PCR (qRT‐PCR) was performed to measure mRNA levels of pro‐inflammatory and anti‐inflammatory cytokines (IL‐1β, TNF‐α, IL‐6, IL‐10, and TGF‐β) as well as tight junction proteins (ZO‐1 and occludin). Gene expression was normalized to the housekeeping genes TATA‐box binding protein (TBP) and glyceraldehyde 3‐phosphate dehydrogenase (GAPDH). qRT‐PCR was conducted using SYBR Green Master Mix (Thermo Fisher Scientific) and gene‐specific primers. Relative gene expression was calculated using the comparative CT (ΔΔCT) method. All samples were analyzed in duplicate to ensure reproducibility. All primers were synthesized by Macrogen (Seoul, Korea). The following primer sets were used for qRT‐PCR analysis. Primer sequences for IL‐1β, TNF‐α, IL‐6, IL‐10, TGF‐β, ZO‐1, occludin, TBP, and GAPDH are listed in Table S2.

### 4.13 Immunofluorescence Staining

Colonic tissues were trimmed at 20 µm and subsequently cryosectioned at a thickness of 7–10 µm using a cryostat. Sections were mounted on glass slides and circled with a hydrophobic barrier using a PAP pen (Abcam). Tissue fixation was performed with 4% PFA for 10 min at room temperature, followed by three washes with ice‐cold PBS (5 min each). Permeabilization was conducted using 0.25% Triton X‐100 (Alfa Aesar) in PBS for 10 min, followed by three additional PBS washes. To block nonspecific binding, sections were incubated for 30 min at room temperature in blocking buffer containing 1% bovine serum albumin (BSA), 22.52 mg/mL glycine, and 0.1% Tween 20 in PBS (PBST).

For immune cell staining, sections were first incubated with FITC‐conjugated anti‐CD4 antibody (RM4‐5, Santa Cruz Biotechnology, Cat# 96127S, 1:100) for 1 h in a humidified chamber at room temperature. After three PBS washes, eFluor 570‐conjugated anti‐Foxp3 antibody (FJK‐16s, Thermo Fisher Scientific/eBioscience, Cat# 41‐5773‐82, 1:40) was applied and incubated overnight at 4°C. Sections were then washed three times with PBS, and nuclei were counterstained with Hoechst 33342 (Invitrogen, 1:2000 in PBS) for 3 min, followed by a final PBS wash. Samples were mounted with 10 µL of aqueous mounting medium. For tight junction protein analysis, additional cryosections were co‐stained with anti‐ZO‐1 antibody (rabbit polyclonal, Invitrogen, Cat# 40–2200, 1:100) and Alexa Fluor 594‐conjugated anti‐Occludin antibody (OC‐3F10, Invitrogen, Cat# 331594, 1:100), both diluted in 1% BSA in PBST. Sections were incubated for 1 h in a humidified chamber at room temperature, followed by three PBS washes. To detect ZO‐1, samples were incubated with Alexa Fluor 488‐conjugated goat anti‐rabbit IgG H&L antibody (goat polyclonal, Abcam, Cat# ab150077, 1:1000) for 1 h at room temperature in the dark. Nuclei were counterstained with Hoechst 33342 (Invitrogen, 1:2000 in PBS) for 3 min, followed by a PBS wash and mounting as described above.

To evaluate the colocalization of Cy5.5‐labeled IBN with CD4^+^ cells in intestinal tissues, additional sections were co‐stained with PE‐conjugated anti‐CD326 antibody (G8.8, BioLegend, Cat# 118205, 1:100) and FITC‐conjugated anti‐CD4 antibody (RM4‐5, Santa Cruz Biotechnology, Cat# 96127S, 1:100), both diluted in 1% BSA in PBST. Sections were incubated for 1 h in a humidified chamber at room temperature, followed by nuclear counterstaining with Hoechst 33342 (Invitrogen, 1:2000 in PBS) for 3 min, PBS wash, and mounting as described above. All fluorescence images were acquired using a Zeiss LSM880 confocal laser scanning microscope.

### 4.14 Flow Cytometry Analysis

For isolation of T cells, entire colons were first washed with cold PBS, cut into 2–3 cm pieces, and incubated in PBS containing 30 mm EDTA on ice for 30 min. After vigorous shaking, tissues were repeatedly washed with PBS until the supernatant became clear. The washed tissues were minced into small pieces and digested in DMEM containing DNase I (2 U/mL, Thermo Fisher Scientific) and Liberase (2.5 mg/mL, Sigma‐Aldrich) at 37°C for 30 min. Digested tissues were passed through a cell strainer into DMEM medium (Welgene) supplemented with 10% fetal bovine serum (FBS). Cells were collected by centrifugation and further purified using a 40%/80% Percoll (Sigma–Aldrich) density gradient. Cells at the interface were harvested, washed with DMEM, and used for flow cytometric analysis.

For flow cytometric analysis, cells were resuspended in 1% BSA and stained with fixable viability dye (eBioscience Fixable Viability Dye eFluor 450, Thermo Fisher Scientific/eBioscience, Cat# 65‐0863‐14, 1:1000) at 4°C for 30 min. Fc receptors were blocked by incubating cells with CD16/CD32 antibody (Rat monoclonal, Thermo Fisher Scientific/eBioscience, Cat# 14‐0161‐85, 1:20) at room temperature for 10 min. Subsequently, cells were stained with APC/Cyanine7‐conjugated anti‐CD45 antibody (Rat monoclonal, Biolegend, Cat# 103115, 1:100), FITC‐conjugated anti‐CD3 antibody (Rat monoclonal, Biolegend, Cat# 100203, 1:100), and APC‐conjugated anti‐CD4 antibody (Rat monoclonal, RM4‐5, Thermo Fisher Scientific/eBioscience, Cat# 17‐0042‐82, 1:100) at 4°C for 30 min. After washing, cells were permeabilized using a commercial kit (eBioscience Foxp3/Transcription Factor Staining Buffer Set, Thermo Fisher Scientific/eBioscience, Cat# 00‐5523‐00) according to the manufacturer's instructions and stained with PE‐conjugated anti‐Foxp3 antibody (Rat monoclonal, FJK‐16s, Thermo Fisher Scientific/eBioscience, Cat# 12‐5773‐82, 1:100) at room temperature for 30 min. After washing, cells were resuspended in 1% BSA and analyzed by flow cytometry (CytoFLEX S, Beckman Coulter, Brea, California, United States, NFEC‐2024‐11‐300452).

### 4.15 In vivo Toxicity Evaluation

Six‐week‐old female C57BL/6 mice were acclimated for 1 week prior to experimentation. Mice were orally administered IBN (60 mg/dose) or PBS on days −6, −4, −2, 0, 2, 4, 6, and 8. Throughout the study period, animals were monitored for changes in body weight and general behavior. At the end of the observation period, blood samples were collected via cardiac puncture and submitted to the Korea Non‐Clinical Technology Support Center for blinded hematological analysis. Mice were then euthanized, and major organs—including the heart, liver, lungs, kidneys, and spleen—were harvested for histopathological evaluation. Tissues were fixed in 4% PFA in PBS, transferred to 70% ethanol, embedded in paraffin, sectioned, and stained with H&E. All histological assessments were performed in a blinded manner to minimize observer bias.

### 4.16 Cell Culture

The HCT‐116 human colonic epithelial cell line was obtained from the American Type Culture Collection (ATCC, Manassas, VA, USA). HCT‐116 cells were cultured in RPMI 1640 medium (Welgene) supplemented with 10% (v/v) heat‐inactivated FBS, 100 U/mL penicillin, 100 µg/mL streptomycin, and 2 mm l‐glutamine at 37°C in a humidified atmosphere containing 5% CO_2_.

### 4.17 In vitro Evaluation of ZO‐1 Expression

HCT‐116 cells were seeded in 6‐well plates and cultured in RPMI 1640 medium (Welgene) supplemented with 10% FBS, 100 U/mL penicillin, and 100 µg/mL streptomycin. Cells were treated with butyrate (0.5 mm) or control medium in the presence or absence of H_2_O_2_ (200 µm) for 24 h. Total RNA was extracted using TRIzol reagent (Thermo Fisher Scientific), and cDNA was synthesized using the amfiRivert cDNA Synthesis Platinum Master Mix (GenDEPOT). mRNA levels of ZO‐1 were quantified by qRT‐PCR as described above.

### 4.18 Quantitative Analysis of Butyrate in Plasma, Feces, And Colon Tissues By GC‐MS

Butyrate measurement in biological samples was performed using gas chromatography‐mass spectrometry (GC‐MS). Plasma, feces, and colon samples were collected from designated experimental groups (DSS (negative control], NaB, inulin, IBN, etc., *n* = 3), and stored at −80°C prior to analysis. For sample preparation, plasma was mixed at a 1:1 ratio (v:v) with 50% methanol. Feces and colon tissues were homogenized with 50% methanol at a ratio of 400 µL per 10 mg sample, feces samples were sonicated for 10 min at 10°C, while colon samples were homogenized for 10 min using an ultrasonic processor (KFS‐150N, KORPROTECH, South Korea) in an ice bath. Samples were kept in −80°C for 12 h for protein precipitation. All samples were centrifuged at 15,000 g for 10 min at 4°C, followed by extraction of 100 µL supernatant mixed with 10 µL of internal standard (2‐ethylbutyric acid). Subsequently, 100 µL of methyl tert‐butyl ether (MTBE) was added, and extraction was performed twice. The upper organic layer was transferred to a tube containing 15 mg MgSO4, centrifuged again at 13,000 g for 10 min at 4°C, and derivatized with N‐tert‐butyldimethylsilyl‐N‐methyltrifluoroacetamide (MTBSTFA, final concentration 10%) at 80°C for 30 min, followed by cooling to ambient temperature for 30 min. For GC‐MS analysis, 1 µL of the derivatized sample was injected into an Agilent 8890 gas chromatograph coupled to a Pegasus BT time‐of‐flight mass spectrometer (LECO,St. Joseph, MI, USA), equipped with an HP‐5MS UI column (30 m × 0.25 mm, 0.25 µm, Agilent Technologies). The injection temperature was 240°C, and helium was used as the carrier gas at 1 mL/min. The GC oven temperature was programmed initially at 50°C for 2 min, increased to 150°C at 5°C/min, then ramped to 300°C at 50°C/min and held for 5 min. Electron ionization (70 eV) was employed, and data acquisition was performed in full‐scan mode (m/z 40–600). Quantification was carried out using external calibration curves established by spiking samples with butyric acid standards at concentrations of 0.5, 1, 5, 10, and 50 µm, each prepared and analyzed in triplicate.

### 4.19 Microbiome Analysis

Fecal samples were collected and submitted to Macrogen Inc. (Seoul, Republic of Korea) for 16S rRNA gene sequencing and microbiome analysis. Genomic DNA was extracted using the DNeasy PowerSoil Pro Kit (Qiagen, Hilden, Germany) according to the manufacturer's instructions. DNA concentration was measured using PicoGreen reagents on a VICTOR Nivo system (PerkinElmer). Sequencing libraries for 16S rRNA gene analysis were prepared following the Illumina 16S Metagenomic Sequencing Library Preparation protocol using 5 ng of input DNA, Herculase II Fusion DNA Polymerase (Agilent Technologies), and universal primers targeting the V3–V4 region. After the first PCR amplification, products were purified with AMPure beads, followed by index PCR using Nextera XT indexed primers. The final libraries were purified, quantified, and quality‐checked using the TapeStation D1000 ScreenTape system, normalized, pooled, and quantified by qPCR before sequencing on the Illumina MiSeq i100 platform. Sequences were curated using the community‐supported software program mothur (v.1.48.3) and by following the steps outlined in the MiSeq SOP (http://www.mothur.org/wiki/MiSeq_SOP). Sequences were assigned to OTUs using a cut‐off value of 0.03 and classified against the Ribosomal Database Project 16S rRNA gene training set (v.19) using a naive Bayesian approach with an 80% confidence threshold. Curated OTU sequence data were converted to relative abundance ± s.e.m. The Shannon diversity and inverse‐Simpson indices were used to calculate alpha diversity, and the Yue and Clayton dissimilarity metric was used for beta diversity measures. Analysis of molecular variance was used to detect significant clustering of different treatment groups in non‐metric multidimensional scaling. To confirm what specific bacterial taxa were over‐ and/or under‐represented among groups, we analyzed relative abundance results by using linear discriminant analysis effect size.

### 4.20 Statistical Analysis

All experiments were independently performed at least twice with technical duplicates. Data are presented as mean ± standard error of the mean (s.e.m.). Statistical significance was assessed using one‐way or two‐way ANOVA followed by the least significant difference (LSD) post hoc test. No data transformation or normalization was performed unless otherwise stated. Data were approximately normally distributed, and variance was similar between groups. The number of independent experimental replicates is indicated in the figure legends, and each figure presents data from a representative independent experiment. No samples were excluded from the analysis. Statistical significance is indicated as ^*^ *P* < 0.05, ^**^ *P* < 0.01, ^***^ *P* < 0.001, and ^****^ *P* < 0.0001. All statistical analyses were performed using GraphPad Prism v8.0 (GraphPad Software).

## Author Contributions

Y.L. supervised the project. N.Y. and Y.L. designed the experiments. N.Y. performed most experiments. B.L., H.‐J.J., J.Y.K and S.P. aided with some experiments. S.K., D.‐K.L. and Y.L. analyzed the data. Y.L. wrote the paper.

## Conflicts of Interest

The authors declare no conflicts of interest.

## Supporting information




**Supporting File**: smll73162‐sup‐0001‐SuppMat.docx.

## Data Availability

The authors declare that data supporting the findings of this study are available within the article and its Supplementary Information files. All relevant data can be provided by the authors upon reasonable request.
