# Supplementary material for: Inulin‐Butyrate Nanogel for Modulation of Gut Microbiome, Intestinal Barrier, and Regulatory T‐Cells in Colitis
Source: Small. 2026 Mar 19;22(27):e13252. doi: 10.1002/smll.202513252 (PMC13173310; doi:10.1002/smll.202513252)
Supplement: Supplementary file 1 — Supporting File: smll73162‐sup‐0001‐SuppMat.docx. [file SMLL-22-e13252-s001.docx]

**Supplementary Information for**

**Inulin-Butyrate Nanogel for Modulation of Gut Microbiome and Regulatory T-cells in Colitis**

**Nayoon Park^1,2^, Bom Lee^1,2^,** **Hyeon-Jeong Jeon^4^, Ji Yeon Kim^3^, Suyeon Park^3^, Seong-Eun Kim^4^, Dong-Kyu Lee^3^, and Yonghyun Lee^1,2^***

^1^College of Pharmacy, Ewha Womans University, Seoul 03760, South Korea

^2^Gradutate Program in Innovative Biomaterials Convergence, Ewha Womans University, Seoul, 03760, South Korea

^3^College of Pharmacy, Chung-Ang University, Seoul 06974, South Korea

^4^Division of Gastroenterology, Department of Internal Medicine, Ewha Womans University Mokdong Hospital, Ewha Womans University College of Medicine, Seoul 03760, South Korea

*E-mail: y.lee@ewha.ac.kr

**SUPPLEMENTARY INFORMATION**

- Supplementary Figure 1. Synthesis of inulin-butyrate conjugate.
- Supplementary Figure 2. Inulin-butyrate Nanogel (IBN) has a gel-like property.
- Supplementary Figure 3. pH-dependent colloidal stability and drug release profiles of IBN.
- Supplementary Figure 4. Quantitative analysis of butyrate in feces and colon tissues by GC-MS.
- Supplementary Figure 5. Inulin has a better therapeutic activity compared to the resistant starch in a DSS-induced colitis model.
- Supplementary Figure 6. IBN exhibits superior therapeutic efficacy compared to butyrate–inulin mixture in DSS-induced colitis.
- Supplementary Figure 7. Therapeutic efficacy of IBN in DSS-induced colitis without preventive treatment.
- Supplementary Figure 8. Safety profiles of IBN.
- Supplementary Figure 9. Flow cytometry gating strategy for CD4+Foxp3+ regulatory T cell analysis.
- Supplementary Table 1. A series of inulin–butyrate conjugates synthesized under various conditions.
- Supplementary Table 2. Primer sequences used for qRT-PCR.

**Supplementary Figures**


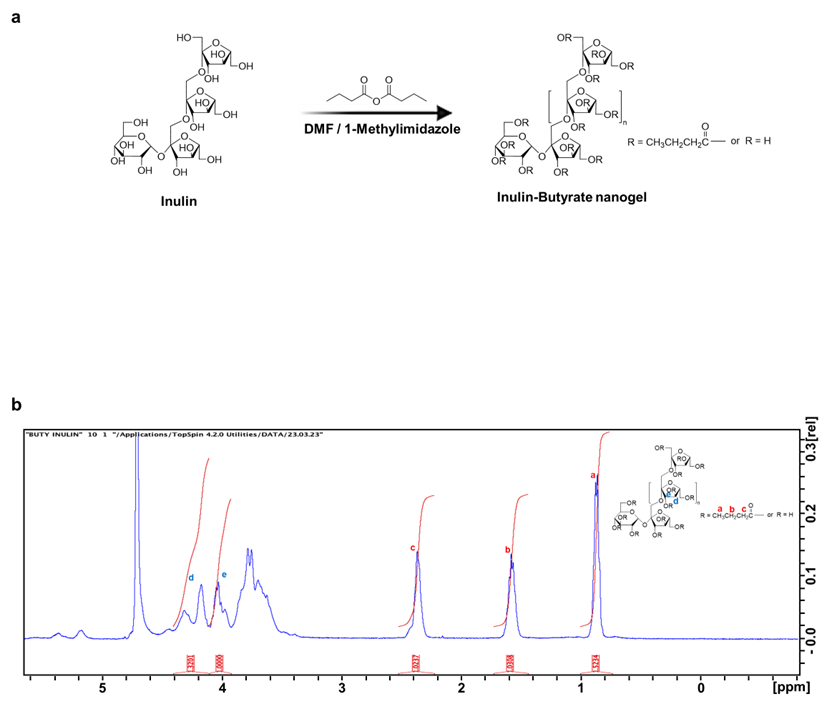


**Supplementary Figure 1. Synthesis of inulin-butyrate conjugate.** **a,** Schematic diagram of the synthesis of inulin–butyrate conjugate. **b,** ¹H NMR spectra of inulin-butyrate conjugate in D₂O. Broad signals between 3.0–5.5 ppm represent the protons of the inulin backbone, while new peaks in the region of 0.8–2.5 ppm correspond to the methyl and methylene protons of conjugated butyrate, indicating successful esterification.


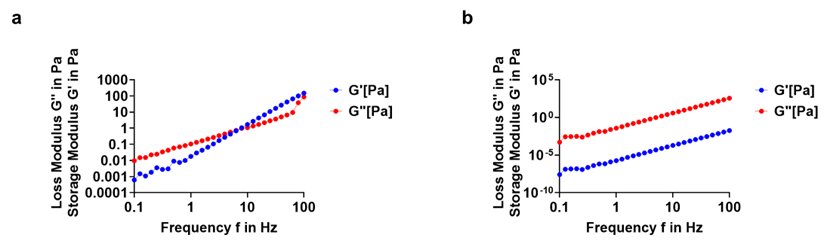


**Supplementary Figure 2**. **Inulin-butyrate Nanogel (IBN) has a gel-like property. a,** Frequency sweep analysis of IBN at the treatment concentration, showing a crossover point where G″ exceeds G′ at low frequencies, followed by predominantly elastic behavior (G′ > G″) at higher frequencies—indicative of viscoelastic gel properties. **b,** Frequency sweep analysis of IBN at the low concentration, with G″ consistently higher than G′, indicating viscous-dominant, fluid-like behavior. G′ represents the elastic (solid-like) modulus and G″ the viscous (liquid-like) modulus. These results highlight the concentration-dependent viscoelasticity of IBN.


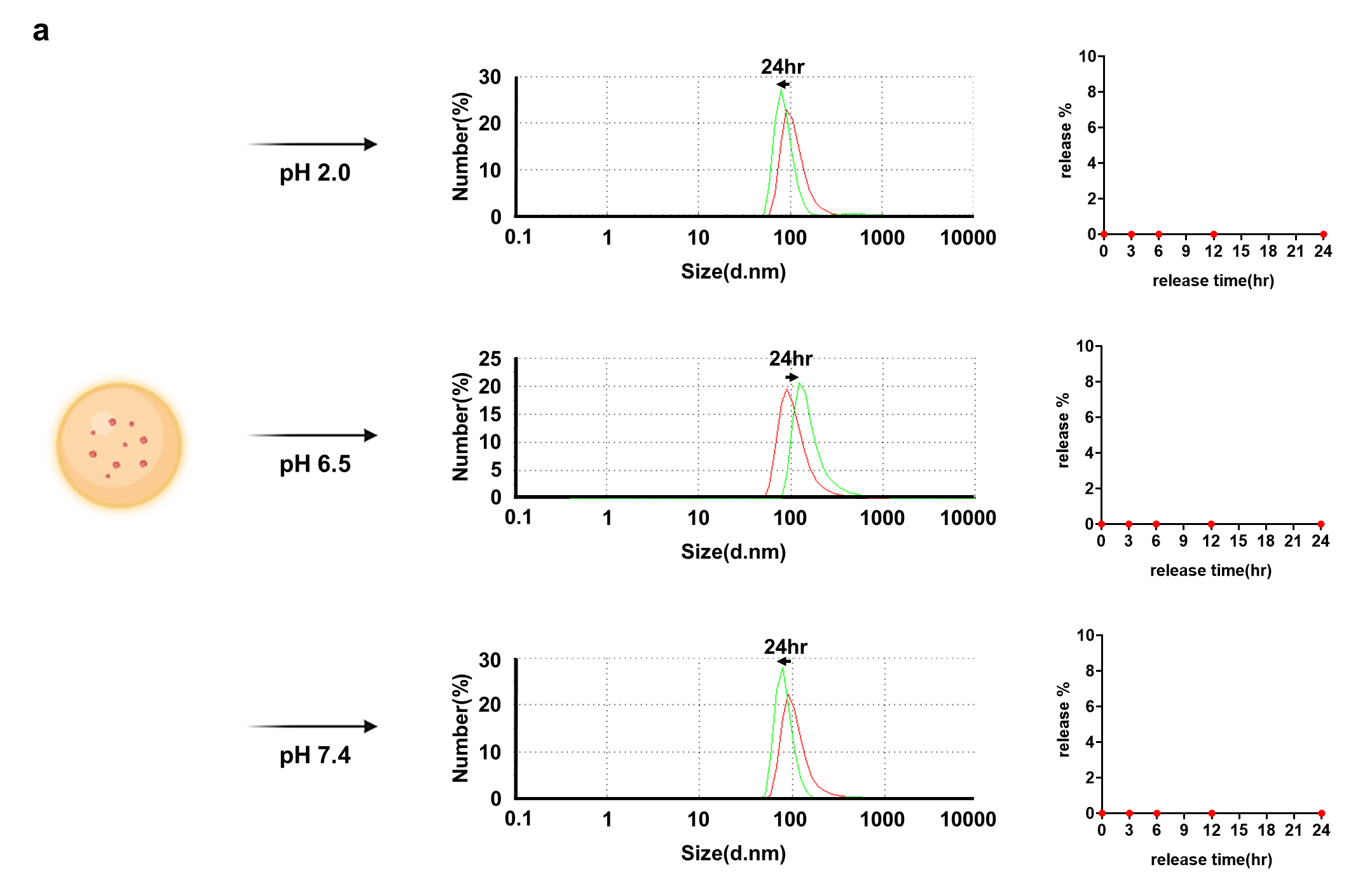


**Supplementary Figure 3**. **pH-dependent colloidal stability and drug release profiles of IBN.** **a,** Schematic representation of IBN nanoparticles. DLS size distribution curves (middle panels) and cumulative drug release profiles (right panels) of IBN at pH 2.0, pH 6.5, and pH 7.4 over 24 hours. Data are presented as mean ± s.e.m. from a representative experiment of three independent experiments.


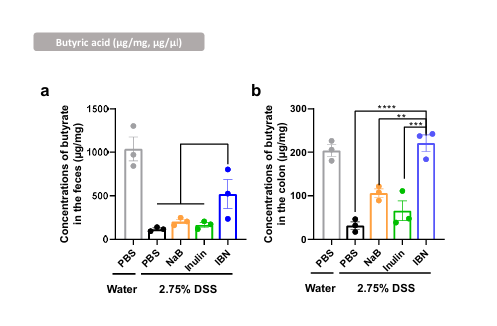


**Supplementary Figure 4**. **Quantitative analysis of butyrate in feces and colon tissues by GC–MS. a,** butyrate levels in feces. **b,** butyrate levels in colon tissues. Feces and colon samples were collected from each treatment group [PBS, NaB, inulin, IBN, etc.; n = 3] and stored at −80 °C until analysis. Samples were extracted with 50% methanol, spiked with 2-ethylbutyric acid as an internal standard, and subjected to liquid–liquid extraction with MTBE. After derivatization with MTBSTFA, butyrate concentrations were quantified using GC–MS (Agilent 8890 GC coupled to Pegasus BT TOF-MS) based on external calibration curves (0.5–50 µM) prepared and analyzed in triplicate.

**
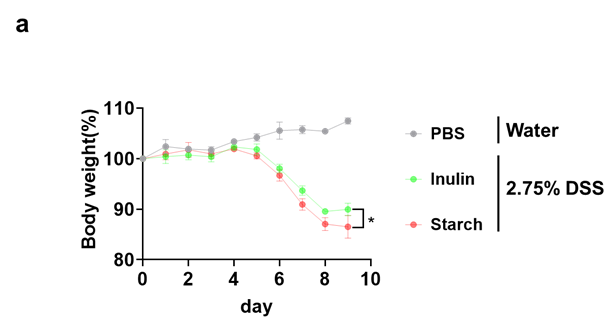
**

**Supplementary Figure 5**. **Inulin has a better therapeutic activity compared to the resistant starch in a DSS-induced colitis model. a,** Body weight changes in mice administered PBS (Healthy control), starch or inulin following induction of colitis with 2.75% DSS for 6 days. Treatments were administered orally on predetermined days (days -6, -4, -2, 0, 2, 4, 6, and 8) throughout the experimental period. Data are presented as mean ± s.e.m. (n = 4 per group). Statistical analysis was performed using one-way ANOVA followed by LSD post hoc test. *P < 0.05, **P < 0.01 vs. DSS group.


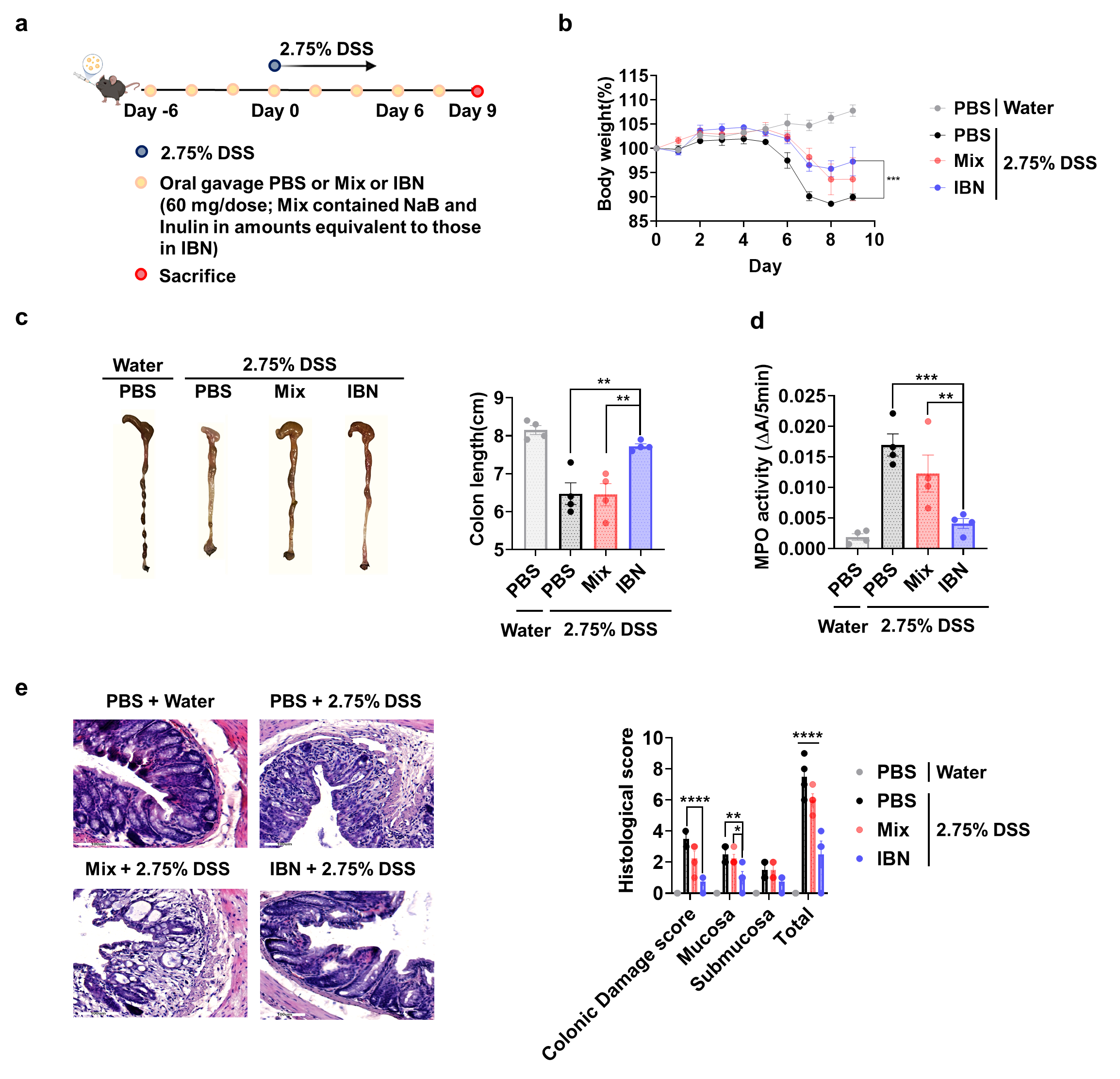


**Supplementary Figure 6**. **IBN exhibits superior therapeutic efficacy compared to butyrate–inulin mixture in DSS-induced colitis. a,** C57BL/6 mice were provided with water or 2.75% DSS-containing water for 6 days. On days -6. -4, -2, 0, 2, 4, 6 and 8 mice were orally administered with PBS, NaB (equivalently to their content in IBN) + Inulin (equivalently to their content in IBN) mix or IBN (60 mg/dose). **b,** Daily bodyweight changes in each group for 9 days. **c-e,** On day 9, animals were euthanized, and (c) colon length, (d) colonic MPO activity, and (e) colonic damage scores were measured. Scale bars, 100 μm (e). Data are presented as mean ± s.e.m. from a representative experiment (n = 4 biologically independent animals) from independent experiments. *p < 0.05, **p < 0.01, ***p < 0.001, ****p < 0.0001, analyzed by (c, d, e) one-way or (b) two-way ANOVA followed by LSD post hoc test.


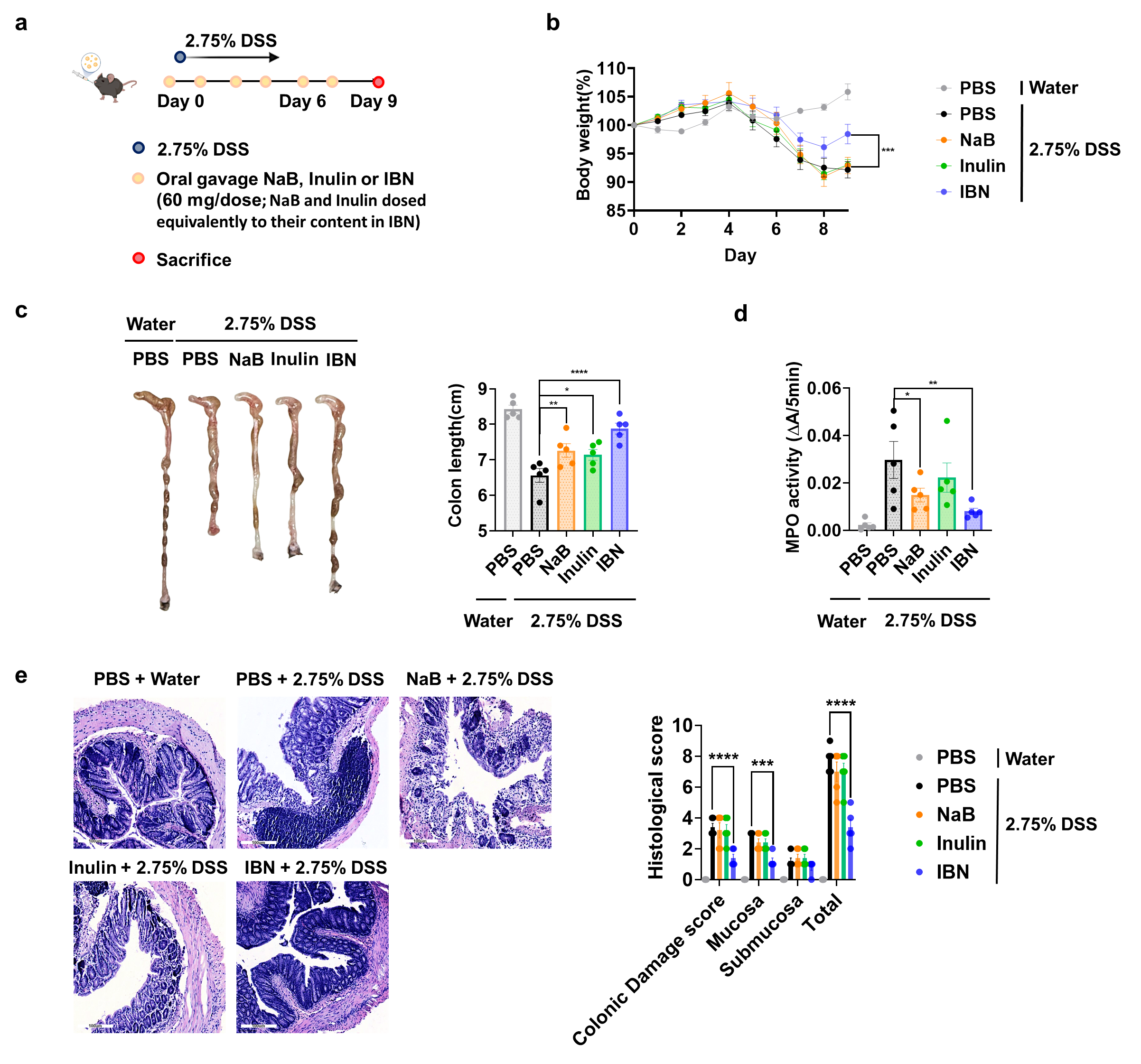


**Supplementary Figure 7** **Therapeutic efficacy of IBN in DSS-induced colitis without preventive treatment. a,** C57BL/6 mice were provided with water or 2.75% DSS-containing water for 6 days. On days 0, 1, 3, 4, 6 and 7 mice were orally administered with PBS, NaB (equivalently to their content in IBN), Inulin (equivalently to their content in IBN) or IBN (60 mg/dose). **b,** Daily bodyweight changes in each group for 9 days. **c-e,** On day 9, animals were euthanized, and (**c**) colon length, (**d**) colonic MPO activity, and (**e**) colonic damage scores were measured. Scale bars, 100 μm (**e**). Data are presented as mean ± s.e.m. from a representative experiment (n = 5 biologically independent animals) from independent experiments. *p < 0.05, **p < 0.01, ***p < 0.001, ****p < 0.0001, analyzed by (**c, d, e**) one-way or (**b**) two-way ANOVA followed by LSD post hoc test.


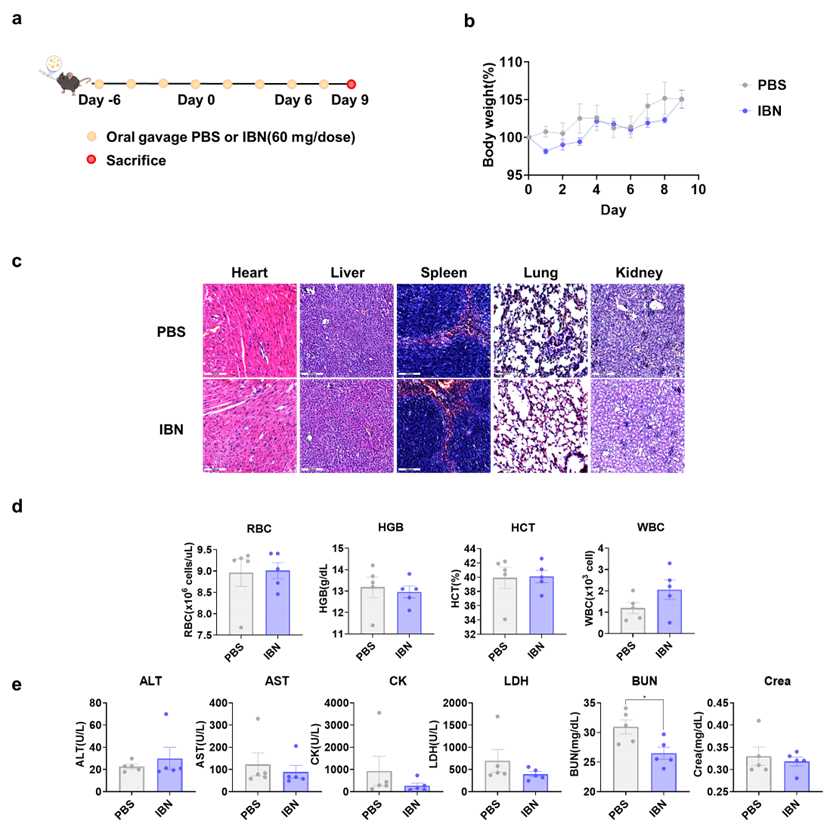


**Supplementary Figure 8. Safety profiles of IBN. a,** C57BL/6 mice were orally administered with PBS or 60 mg/dose of IBN on days -6. -4, -2, 0, 2, 4, 6 and 8. **b,** Daily bodyweight changes in each group for 16 days. **c,** Major organ (heart, liver, kidney, lung, and spleen) sections stained with H&E were analyzed for systemic toxicity evaluation. **d-e,** Blood were analyzed using blood hematology and blood chemistry for systemic toxicity evaluation. Scale bars, 100 μm (c). Data are presented as mean ± s.e.m. from a representative experiment (n = 5 biologically independent animals) from independent experiments. *p < 0.05, **p < 0.01, ***p < 0.001, ****p < 0.0001, analyzed by (d, e) one-way or (b) two-way ANOVA followed by LSD post hoc test.


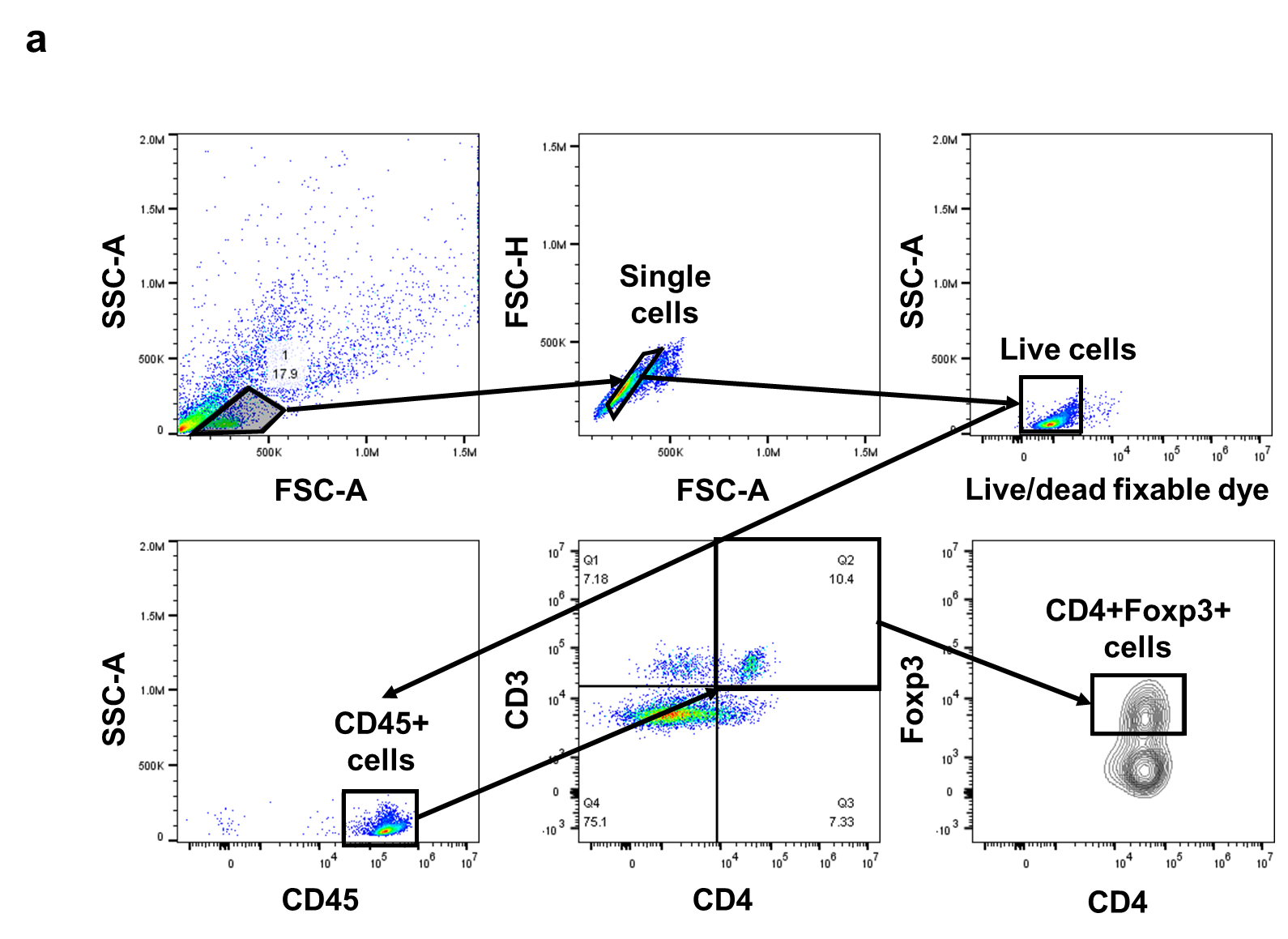


**Supplementary Figure 9. Flow cytometry gating strategy for CD4+Foxp3+ regulatory T cell analysis. a,** Cells were first gated based on FSC-A and SSC-A, followed by doublet exclusion using FSC-H versus FSC-A. Live cells were identified by exclusion of viability dye–positive cells. Immune cells were defined as CD45⁺ cells, and T cells were identified as CD3⁺CD4⁺ cells. Regulatory T cells were defined as Foxp3⁺ cells within the CD3⁺CD4⁺ population. Quantification was performed as the ratio of CD4⁺Foxp3⁺ cells to total CD3⁺ T cells.

|  | Condition 1  (Original) | Condition 2  (x 0.5) | Condition 3  (x 2) | Condition 4  (x 4) |
| --- | --- | --- | --- | --- |
| Inulin | 5mg | 5mg | 5mg | 5mg |
| 1-Methylimidazole | 0.895 ul | 0.448 ul(*0.5) | 1.79 ul(*2) | 3.58 ul(*4) |
| Butyric anhydride | 2.35 ul | 1.175 ul(*0.5) | 4.7 ul(*2) | 9.4 ul(*4) |
| DS Value | 0.51 | 0.32 | 0.72 | - |
| Solubility | High | High | Low | Very low |
| Drug Loading Capacity | 22.71 % | 15.28 % | 29.84 % | - |

**Supplementary Table 1.** A series of inulin–butyrate conjugates synthesized under various conditions.

| Gene  (species) | Forward sequence (5’-3’) | Reverse sequence (5’-3’) |
| --- | --- | --- |
| IL-1β  (mouse) | TGCCACCTTTTGACAGTGATG | ATGTGCTGCTGCGAGATTTG |
| TNF-α  (mouse) | AGGCACTCCCCCAAAAGATG | TTTGCTACGACGTGGGCTAC |
| IL-6  (mouse) | CTTCTTGGGACTGATGCT | CTGGCTTTGTCTTTCTTGTT |
| IL-10  (mouse) | GCTCTTACTGACTGGCATGAG | CGCAGCTCTAGGAGCATGTG |
| TGF-β  (mouse) | TGACGTCACTGGAGTTGTACGG | GGTTCATGTCATGGATGGTGC |
| ZO-1  (mouse) | CTTCTCTTGCTGGCCCTAAAC | TGGCTTCACTTGAGGTTTCTG |
| ZO-1  (human) | GAATGATGGTTGGTATGGTGCG | TCAGAAGTGTGTCTACTGTCCG |
| Occludin  (mouse) | TGAAAGTCCACCTCCTTACAGA | CCGGATAAAAAGAGTACGCTGG |
| TBP  (mouse) | ACCGTGAATCTTGGCTGTAAAC | GCAGCAAATCGCTTGGGATTA |
| GAPDH  (human) | GGAAGCTTGTCATCAATGGAAATC | TGATGACCCTTTTGGCTCCC |

**Supplementary Table 2.** Primer sequences used for qRT-PCR.
